# mRNA‐Lipid Nanoparticle‐Mediated Restoration of PTPN14 Exhibits Antitumor Effects by Overcoming Anoikis Resistance in Triple‐Negative Breast Cancer

**DOI:** 10.1002/advs.202309988

**Published:** 2024-06-21

**Authors:** Wei Li, Masha Huang, Zhaoping Wu, Yu Zhang, Ying Cai, Juncheng Su, Jia Xia, Fan Yang, Desheng Xiao, Wen Yang, Yingjie Xu, Zhaoqian Liu

**Affiliations:** ^1^ Department of Clinical Pharmacology Hunan Key Laboratory of Pharmacogenetics and National Clinical Research Center for Geriatric Disorders Xiangya Hospital Central South University Changsha 410008 P. R. China; ^2^ Institute of Clinical Pharmacology Engineering Research Center for applied Technology of Pharmacogenomics of Ministry of Education Central South University Changsha 410078 P. R. China; ^3^ Department of Biochemistry and Molecular Cell Biology Shanghai Key Laboratory for Tumor Microenvironment and Inflammation Shanghai Jiao Tong University School of Medicine Shanghai 200025 P. R. China; ^4^ Department of Neurosurgery Xiangya Hospital Central South University Changsha 410008 P. R. China; ^5^ Department of Gastrointestinal Surgery Renji Hospital Shanghai Jiao Tong University School of Medicine Shanghai 200127 P. R. China; ^6^ Department of Nephrology Renji Hospital Shanghai Jiao Tong University School of Medicine Shanghai 200127 P. R. China; ^7^ Department of Physiology School of Basic Medical Sciences Shandong University Jinan 250011 P. R. China; ^8^ Department of Pathology School of Basic Medicine Xiangya Hospital Central South University Changsha 410013 P. R. China; ^9^ Key Laboratory of Cell Differentiation and Apoptosis of Chinese Ministry of Education Shanghai Jiao Tong University School of Medicine Shanghai 200025 P. R. China

**Keywords:** anoikis resistance, cancer therapy, mRNA therapeutics, PTPN14, triple‐negative breast cancer

## Abstract

Triple‐negative breast cancer (TNBC) poses a challenging prognosis due to early metastasis driven by anoikis resistance. Identifying crucial regulators to overcome this resistance is vital for improving patient outcomes. In this study, a genome‐wide CRISPR/Cas9 knockout screen in TNBC cells has identified tyrosine‐protein phosphatase nonreceptor type 14 (PTPN14) as a key regulator of anoikis resistance. PTPN14 expression has shown a progressive decrease from normal breast tissue to metastatic tumors. Overexpressing PTPN14 has induced anoikis and inhibited cell proliferation in TNBC cells, while normal human breast cells are unaffected. Mechanistically, PTPN14 is identified as a key factor in dephosphorylating breast cancer antiestrogen resistance 3, a novel substrate, leading to the subsequent inhibition of PI3K/AKT and ERK signaling pathways. Local delivery of in vitro transcribed PTPN14 mRNA encapsulated by lipid nanoparticles in a TNBC mouse model has effectively inhibited tumor growth and metastasis, prolonging survival. The study underscores PTPN14 as a potential therapeutic target for metastatic TNBC, with the therapeutic strategy based on mRNA expression of PTPN14 demonstrating clinical application prospects in alleviating the burden of both primary tumors and metastatic disease.

## Graphical abstract

Through a genome‐wide CRISPR/Cas9 knockout screen, tyrosine‐protein phosphatase nonreceptor type 14 (PTPN14) is identified as a key regulator of anoikis resistance in triple‐negative breast cancer (TNBC). Overexpressing PTPN14 inhibits anoikis resistance, tumorigenicity, and metastasis in TNBC cells by dephosphorylating breast cancer antiestrogen resistance 3, affecting the PI3K/AKT and ERK pathways. In a mouse model, PTPN14 mRNA‐lipid nanoparticle delivery has reduced tumor growth and metastasis, highlighting its therapeutic potential.

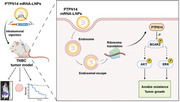

## 1 Introduction

Breast cancer, now the most prevalent cancer globally, includes a subtype known as triple‐negative breast cancer (TNBC), which lacks estrogen receptor and progesterone receptor expression, as well as human epidermal growth factor receptor 2 (HER2) amplification, comprising 24% of new breast tumors.^[^ ^1^ ^]^ TNBC, marked by tumor heterogeneity and a lack of targeted hormone receptors, faces a grim prognosis and lacks effective therapies.^[^ ^2^ ^]^ Its aggressive nature results in common early metastatic relapse and reduced survival rates. Anoikis, a form of apoptosis linked to extracellular matrix detachment,^[^ ^3^ ^]^ develops resistance in breast cancer progression, contributing to metastatic recurrence.^[^ ^4^ ^]^ Addressing anoikis resistance in TNBC provides a promising avenue to inhibit metastatic relapse.

The lack of robust cell and animal models for studying cancer metastasis has been a challenge, but clustered regularly interspaced short palindromic repeats (CRISPR)/Cas9 screens have emerged as advanced tools to address this limitation.^[^ ^5^ ^]^ To identify therapeutic targets crucial for the induction of anoikis, we conducted a genome‐wide CRISPR/Cas9 knockout screening in TNBC cells under ultralow‐attachment (ULA) conditions, mimicking the detachment scenario from the primary site. This led to the discovery of tyrosine‐protein phosphatase nonreceptor type 14 (PTPN14) as a potential factor associated with anoikis. PTPN14 is a nonreceptor protein tyrosine phosphatase that participates in various cancer‐related signaling cascades through its enzymatic activity or protein interactions,^[^ ^6^ ^]^ and has been confirmed to have anticancer effects in several solid tumors.^[^ ^7^ ^]^ PTPN14 promotes the lysosomal degradation of epidermal growth factor receptor (EGFR) by dephosphorylating PKCδ, thereby inhibiting the excessive activation of EGFR signaling.^[^ ^8^ ^]^ Furthermore, PTPN14 forms a protein complex with YAP to inhibit YAP's nuclear translocation, thereby suppressing the transcriptional activation of downstream oncogenic effector factors.^[^ ^9^ ^]^ However, PTPN14 has also been reported to promote gastric cancer progression by initiating the PI3K/AKT/mTOR pathway.^[^ ^10^ ^]^ Therefore, the role of PTPN14 in tumors remains controversial and unclear.

In the last 10 years, significant technological advancements have paved the way for messenger RNA (mRNA) to emerge as a highly promising platform for vaccine development, protein replacement therapy, and genome engineering.^[^ ^11^ ^]^ In vitro transcribed (IVT) mRNA poses no risk of integration into the patient's genome and can naturally degrade in vivo, exhibiting a favorable safety profile. Furthermore, designed IVT mRNA can be rapidly synthesized and produced in vitro without complex preparation processes.^[^ ^12^ ^]^ Currently, mRNA therapy is widely applied in the field of cancer treatment, including mRNA cancer vaccines, mRNA‐encoding cytokines, and chimeric antigen receptors, showcasing its versatility.^[^ ^11^ ^]^ It also offers a straightforward method to restore tumor‐suppressive proteins in several tumor models,^[^ ^13^ ^]^ thereby validating the clinical promise of these proteins in cancer treatment.

In the present study, we established the pivotal role of PTPN14 in TNBC anoikis and tumorigenicity. Additionally, we identified breast cancer antiestrogen resistance 3 (BCAR3) as a novel substrate responsible for the suppression of the PI3K/AKT and ERK pro‐survival pathways by PTPN14. Importantly, we evaluated PTPN14 mRNA therapy in a spontaneous breast cancer metastasis model, highlighting its tumor‐suppressive effects and clinical translational potential.

## 2 Result

### 2.1 A Genome‑Wide Pooled sgRNA Library Screen in a TNBC Cell Anoikis Model

**Figure 1:**
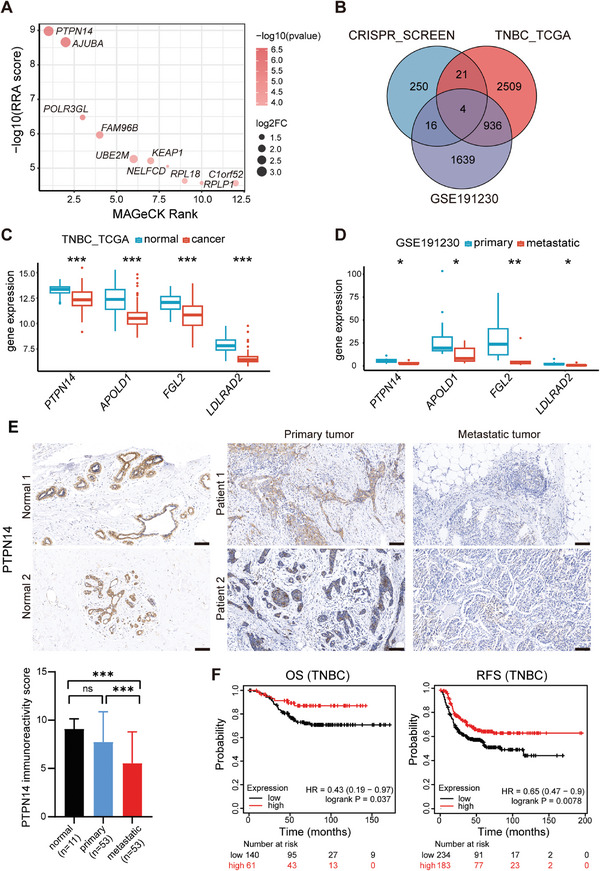
A pooled genome‐wide CRISPR screen in a TNBC anoikis model. A) Genes related to anoikis resistance identified through a genome‐wide CRISPR/Cas9 positive selection screen were ranked based on MAGeCK analysis results, with the top ten genes listed. B) The overlap of the indicated datasets. C) *PTPN14*, *APOLD1*, *FGL2*, and *LDLRAD2* mRNA expression levels in 113 breast cancer adjacent samples and 115 TNBC samples from TCGA database. Student's *t*‐test, ^***^ *p* < 0.001. D) The mRNA expression levels of *PTPN14*, *APOLD1*, *FGL2*, and *LDLRAD2* were assessed in 13 primary breast tumor samples and seven distant metastasis tumor samples from dataset GSE191230 obtained from the GEO database. Student's *t*‐test, ^*^ *p* < 0.05, ^**^ *p* < 0.01. E) Representative IHC images (above) and semiquantitative evaluation of PTPN14 IHC expression (below) in paired primary tumors and metastatic tumors (*n* = 53), and normal breast tissue (*n* = 11). Dunn's multiple comparisons test following Kruskal–Wallis test, ^***^ *p* < 0.001. Scale bar: 100 µm. (F) OS (*n* = 201, low expression: 140; high expression: 61) and RFS (*n* = 417, low expression: 234; high expression: 183) were summarized in the high and low PTPN14 expression groups by using Kaplan–Meier survival curves in TNBC. Log‐rank test, with *p* < 0.05 indicating statistical significance.

To identify the essential genes linked to anoikis resistance in TNBC, we designed a genome‐wide CRISPR/Cas9 knockout screen targeting anoikis (Figure S1A, Supporting Information). GeCKOv2 CRISPR libraries were introduced into the human TNBC cell line MDA‐MB‐231 to generate MDA‐MB‐231 GeCKO cells. Subsequently, MDA‐MB‐231 GeCKO cells were cultured in either normal‐ or ULA plates for 7 days, conducting a positive screen. We hypothesized that the loss of key genes associated with anoikis resistance could aid TNBC cells in evading detachment‐induced apoptosis. Therefore, under ULA culture conditions, cells harboring sgRNAs targeting genes related to anoikis resistance are preferentially selected, leading to the enrichment of their corresponding sgRNAs within the library. Genomic DNA from both the cell groups was extracted, PCR‐amplified, and sequenced. Through Model‐based Analysis of Genome‐wide CRISPR/Cas9 Knockout (MAGeCK) analysis, 291 genes were identified as significantly positively selected (positive *p*‐value < 0.05, positive fold change >1.5, and sgRNAs >3). These 291 genes were ranked based on robust ranking aggregation (RRA) scores and *p*‐values. **Figure** **1**A shows the top ten ranked genes.

Considering that the loss of the key gene we identified favored anoikis resistance in breast cancer cells, it was most likely to possess tumor‐suppressive activity and was downregulated in metastatic sites compared to the primary site. We employed The Cancer Genome Atlas (TCGA)‐TNBC and GSE191230 cohorts to compare differential gene expression between normal and cancer samples, as well as primary and metastatic samples, utilizing |log2FC| > 0.58 (fold change >1.5) and *p*‐value < 0.05. The differentially expressed genes (DEGs) identified from these cohorts intersected with the positive genes from the CRISPR‐based screen, resulting in a final set of four genes: *PTPN14*, *APOLD1*, *FGL2*, and *LDLRAD2* (Figure 1B).

Among these, *PTPN14* exhibited the highest expression in normal samples (Figure 1C) and its expression in metastatic samples was lower than that in primary samples (Figure 1D). Furthermore, in the results of the MAGeCK analysis, *PTPN14* displayed significantly higher RRA scores, *p* values, and log2FC values when contrasted with *APOLD1*, *FGL2*, and *LDLRAD2*, and *PTPN14* ranked first in the MAGeCK Rank (Figure S1B, Supporting Information). Therefore, we hypothesized that *PTPN14* is likely to be a key gene involved in breast cancer anoikis resistance. To validate the changes in PTPN14 expression during breast cancer progression, we performed immunohistochemistry (IHC) analysis to compare PTPN14 expression in 11 normal breast tissues and 53 pairs of matched primary and metastatic breast cancer tissues. The 53 breast cancer patients were all in stage III or higher, with 12 cases being TNBC (Table S1, Supporting Information). The results indicated significant differences in the expression levels of PTPN14 among these three types of tissues, with normal breast tissues and primary breast cancer lesions exhibiting higher expression than metastatic breast cancer lesions (Figure 1E; Figure S1C, Supporting Information). Although the mean PTPN14 immunoreactivity score in normal breast tissues appeared to be higher than in primary breast cancer lesions based on the statistical graph, the difference did not reach statistical significance. We believe this is partly due to the small sample size in this group. Therefore, we examined the protein expression levels of PTPN14 in normal breast tissue and primary breast tumor in a larger sample size using the UALCAN online platform (https://ualcan.path.uab.edu/analysis‐prot.html). The result was consistent with mRNA expression data from the TCGA database. PTPN14 protein expression level was higher in normal breast tissue compared to the primary breast tumor (Figure S1D, Supporting Information). Furthermore, we used the online tool Kaplan–Meier Plotter (https://kmplot.com/analysis/) to conduct a Kaplan–Meier survival analysis. In patients with TNBC, elevated PTPN14 mRNA expression was correlated with improved overall survival (OS) (*n* = 201, low expression:140; high expression:61, log‐rank *p* = 0.037) and relapse‐free survival (RFS) (*n* = 417, low expression:234; high expression:183, log‐rank *p* = 0.0078; Figure 1F). These results provide additional evidence for the potential role of PTPN14 in TNBC progression.

### 2.2 PTPN14 Knockout Promoted Anoikis Resistance and In Vivo Tumorigenicity

**Figure 2:**
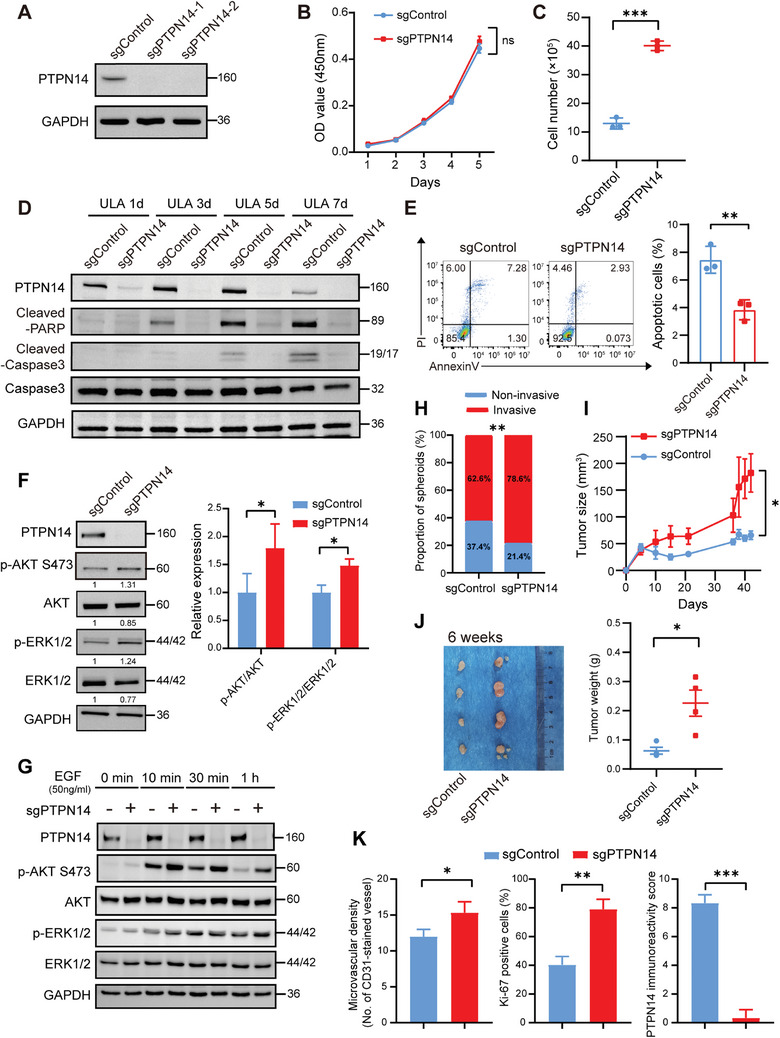
PTPN14 knockout promoted anoikis resistance and in vivo tumorigenicity in TNBC cells. A) Validation of PTPN14 knockout in MDA‐MB‐231 cells through Western Blotting. Three independent experiments were performed. B) Cell proliferation of the control and PTPN14‐KO MDA‐MB‐231 cells in monolayer adherent culture was assessed every 24 h for 5 days using the CCK‐8 assay (*n* = 3). Student's *t*‐test, with *p* < 0.05 indicating statistical significance. C) The counts of living cells for both control MDA‐MB‐231 cells and PTPN14‐KO MDA‐MB‐231 cells were determined after culturing under ULA conditions for 7 days (*n* = 3). Student's *t*‐test, ^***^ *p* < 0.001. D) Assessing the expression levels of apoptotic proteins in control MDA‐MB‐231 cells and PTPN14‐KO MDA‐MB‐231 cells after culturing under ULA conditions for 1 day, 3 days, 5 days, and 7 days. Three independent experiments were performed. E) After culturing for 3 days under ULA conditions, cell apoptosis was measured by flow cytometry in both control MDA‐MB‐231 cells and PTPN14‐KO MDA‐MB‐231 cells. Left, representative flow cytometry result plots; right, statistical summaries of the results from three independent experiments. Student's *t*‐test, ^**^ *p* < 0.01. F) Western Blot analysis was performed to assess the phosphorylation of AKT and ERK in PTPN14‐KO MDA‐MB‐231 cells after 12 h of culture under ULA condition (left), along with statistical summaries of the results from three independent experiments (right). Student's *t*‐test, ^*^ *p* < 0.05. G) PTPN14‐KO and control MDA‐MB‐231 cells were serum‐starved for 24 h and stimulated with EGF for the indicated time, and Western Blot analysis was performed to assess their AKT and ERK phosphorylation. Three independent experiments were performed. H) Quantification of two types of clonal spheroids in the 3D invasion analysis of control and PTPN14‐KO MDA‐MB‐231 cells (*n* = 3). Student's *t*‐test, ^**^ *p* < 0.01. I) The tumor growth curves of the sgControl group and the sgPTPN14 group (*n* = 4). Student's *t*‐test, ^*^ *p* < 0.05, error bars represent the SEM. J) After 6 weeks, the tumors formed by control MDA‐MB‐231 cells and PTPN14‐KO MDA‐MB‐231 cells were excised, photographed (left) and weighed (right) (*n* = 4). Student's *t*‐test, ^*^ *p* < 0.05, error bars represent the SEM. K) Quantification of CD31, Ki‐67, and PTPN14 IHC staining results of two groups of tumor sections (*n* = 3). Student's *t*‐test, ^*^ *p* < 0.05, ^**^ *p* < 0.01, ^***^ *p* < 0.001.

To validate our genome‐wide CRISPR/Cas9 knockout screen results and to investigate the effect of PTPN14 deletion on anoikis resistance in TNBC cells, we employed CRISPR/Cas9 technology to individually knock out PTPN14 in MDA‐MB‐231 and BT549 cells, both being human TNBC cell lines. Western Blot analysis and genomic DNA sequencing confirmed the successful depletion of PTPN14 in both cell lines (**Figure** **2**A; Figure S2A,B, Supporting Information). Using Cell Counting Kit‐8 (CCK‐8) and colony formation assays, we observed that the PTPN14 knockout did not significantly affect the proliferation of MDA‐MB‐231 cells under normal monolayer culture conditions, although it had a minor effect on BT549 cells (Figure 2B; Figure S2C,E, Supporting Information). However, under ULA conditions, the PTPN14‐knockout (PTPN14‐KO) group exhibited a significantly higher number of viable cells than the control group after seven days of cultivation (Figure 2C; Figure S2D, Supporting Information). These findings are consistent with the results of our CRISPR/Cas9 knockout screening.

To further demonstrate that PTPN14 knockout affected anoikis resistance in breast cancer cells, we analyzed changes in the expression of apoptosis‐related proteins in PTPN14‐KO and control cells under ULA conditions. The silencing of PTPN14 hindered apoptosis, leading to a reduction in caspase‐3 cleavage and that of its substrate PARP. Additionally, PTPN14 expression declined as the culture duration increased under ULA conditions (Figure 2D). Consistent with the Western Blot findings, cell apoptosis assessed through flow cytometry also demonstrated that after 3 days of ULA culture, the PTPN14‐KO group had a lower percentage of apoptotic cells than the control group, indicating a reduced occurrence of cell apoptosis (Figure 2E).

Activation of the PI3K/AKT and ERK signaling pathways is the most crucial mechanism by which tumor cells attain anoikis resistance.^[^ ^14^ ^]^ Therefore, we focused on alterations in AKT and ERK1/2 phosphorylation. Notably, PTPN14‐KO breast cancer cells showed higher phosphorylation levels of AKT Ser473 and ERK 1/2 than the control cells after 12 h of culture under ULA conditions (Figure 2F; Figure S2F, Supporting Information). To gain a further understanding of the effect of PTPN14 on downstream pro‐survival signals, we investigated how PTPN14 knockout affects the phosphorylation of AKT and ERK1/2 in MDA‐MB‐231 cells following epidermal growth factor (EGF) stimulation. There is ample evidence to suggest that once the EGF receptor is ligand‐bound, it activates multiple well‐defined signaling pathways, including Ras/ERK and PI3K/AKT. Compared to the control cells, the phosphorylation level of AKT Ser473 was significantly increased in PTPN14 knockout cells. No significant difference in the phosphorylation level of ERK1/2 was observed between PTPN14 knockout cells and control cells (Figure 2G). These results suggested that PTPN14 plays a significant role in anoikis resistance.

To assess the implications of the PI3K/AKT and ERK signaling pathways on the anoikis resistance function of PTPN14‐KO cells, we used MK‐2206 and GSK1120212, which are inhibitors of AKT and ERK respectively, to treat PTPN14‐KO MDA‐MB‐231 cells. Specifically, the PTPN14‐KO cells in the inhibitor treatment groups were cultured under ULA condition until day 3, then treated with MK‐2206 at a final concentration of 20 µm for 48 h, or cultured under ULA condition until day 4, then treated with GSK1120212 at a final concentration of 250 nm for 24 h. Compared to untreated PTPN14‐KO cells cultured under ULA conditions for 5 days, the inhibitor‐treated PTPN14‐KO cells showed increased expression of cleaved‐PARP (Figure S2G,H, Supporting Information). This suggests that treatment with AKT and ERK inhibitors increases cell apoptosis of PTPN14‐KO cells under anoikis conditions, impairing anoikis resistance.

The metastatic process of breast cancer cells requires not only anoikis resistance but also a high degree of invasiveness. A previous study has reported that PTPN14 suppresses metastatic properties of TNBC cells through cooperating with KIBRA.^[^ ^15^ ^]^ Therefore, we used 3D invasion assays to assess the changes in cell invasiveness after altering PTPN14 expression. Notably, PTPN14 knockout significantly enhanced the number of invasive clonal spheroids and the number of protrusions at the cell edges (Figure 2H; Figure S2I, Supporting Information).

Activation of the PI3K/AKT and ERK signaling pathways promotes growth in tumors.^[^ ^16^ ^]^ Meanwhile, cells grown under detached conditions closely mimic the in vivo tumor microenvironment compared with cells grown in monolayers.^[^ ^17^ ^]^ Therefore, we investigated whether PTPN14 depletion affected tumor growth. Female BALB/c nude mice were randomly allocated into two groups and injected with distinct MDA‐MB‐231 stable transfectants into the right mammary fat pad to induce orthotopic tumor formation. One group received PTPN14 knockout cells (sgPTPN14), whereas the other group received the corresponding control cells (sgControl). At different time points post‐injection, the measurement data of tumor size showed that the tumor growth rate in the sgPTPN14 group was faster than that in the sgControl group (Figure 2I). Approximately 6 weeks after injection, the mice were sacrificed to extract orthotopic tumors. Tumor volume and weight in the sgPTPN14 group were significantly higher than those in the sgControl group (Figure 2J). There were no noticeable differences in body weights between the two groups of mice (Figure S2J, Supporting Information). Consistent with this observation, histological examination of tumor sections indicated widespread necrosis in the sgPTPN14 group tumors (dashed line annotation), in contrast to the absence of necrosis in the sgControl group tumors, and the substantial presence of residual Matrigel, indicating a significantly accelerated growth rate in the sgPTPN14 group compared to that in the sgControl group. Additionally, tumors in the sgPTPN14 group exhibited greater vascularization as indicated by CD31 staining, demonstrating the formation of more microvascular structures. There was also a higher proportion of cells expressing the proliferation marker Ki‐67 in the sgPTPN14 group compared to that of the sgControl group (Figure 2K; Figure S2K, Supporting Information). Taken together, the results of the in vitro experiments suggest that the PTPN14 knockout enhances anoikis resistance, while the in vivo experiments confirm that the PTPN14 knockout promotes tumorigenicity in breast cancer cells.

### 2.3 Overexpression of PTPN14 Inhibited Anoikis Resistance, In Vivo Tumorigenicity and Lung Metastasis

**Figure 3:**
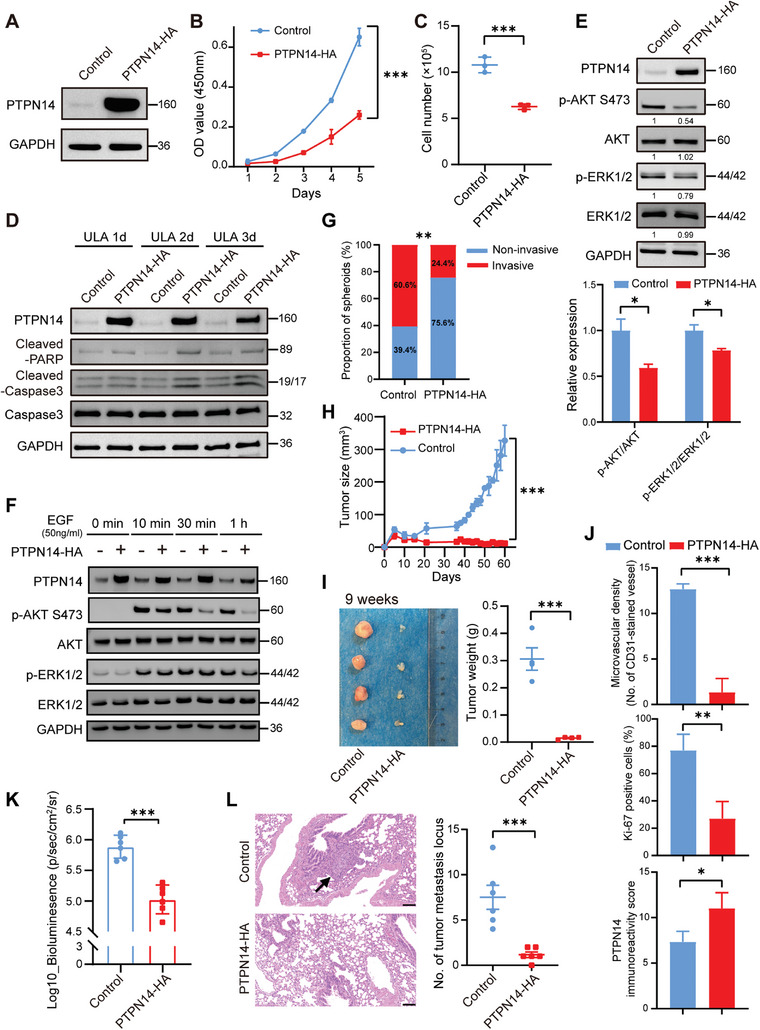
PTPN14 overexpression induced anoikis and suppressed in vivo tumorigenicity and pulmonary metastasis in TNBC cells. A) Validation of PTPN14 overexpression in MDA‐MB‐231 cells through Western Blotting. Three independent experiments were performed. B) Cell proliferation of the control and PTPN14‐OE MDA‐MB‐231 cells in monolayer adherent culture was assessed every 24 h for 5 days using the CCK‐8 assay (*n* = 3). Student's *t*‐test, ^***^ *p* < 0.001. C) The counts of living cells for both control MDA‐MB‐231 cells and PTPN14‐OE MDA‐MB‐231 cells were determined after culturing under ULA conditions for 7 days (*n* = 3). Student's *t*‐test, ^***^ *p* < 0.001. D) Assessing the expression levels of apoptotic proteins in control MDA‐MB‐231 cells and PTPN14‐OE MDA‐MB‐231 cells after culturing under ULA conditions for 1, 2, and 3 days. Three independent experiments were performed. (E) Western Blot analysis was performed to assess the phosphorylation of AKT and ERK in PTPN14‐OE MDA‐MB‐231 cells after 12 h of culture under ULA condition (above), along with statistical summaries of the results from three independent experiments (below). Student's *t*‐test, ^*^ *p* < 0.05. F) PTPN14‐OE and control MDA‐MB‐231 cells were serum‐starved for 24 h and stimulated with EGF for the indicated time, and Western Blot analysis was performed to assess their AKT and ERK phosphorylation. Three independent experiments were performed. G) Quantification of two types of clonal spheroids in the 3D invasion analysis of control and PTPN14‐OE MDA‐MB‐231 cells (*n* = 3). Student's *t*‐test, ^**^ *p* < 0.01. (H) The tumor growth curves of the control group and the PTPN14‐HA group (*n* = 4). Student's *t*‐test, ^***^ *p* < 0.001, error bars represent the SEM. I) After 9 weeks, the tumors formed by control MDA‐MB‐231 cells and PTPN14‐OE MDA‐MB‐231 cells were excised, photographed (left), and weighed (right) (*n* = 4). Student's *t*‐test, ^***^ *p* < 0.001, error bars represent the SEM. J) Quantification of CD31, Ki‐67, and PTPN14 IHC staining results of two groups of tumor sections (*n* = 3). Student's *t*‐test, ^*^ *p* < 0.05, ^**^ *p* < 0.01, ^***^ *p* < 0.001. K) Quantification of bioluminescence intensity of ex vivo lung imaging (*n* = 6). Student's *t*‐test, ^***^ *p* < 0.001. L) Representative images of H&E staining in lung tissue sections from two groups of mice (left), along with counting and statistical analysis of tumor metastatic sites (right) (*n* = 6). The black arrow indicates the metastatic site. Scale bar: 100 µm. Student's *t*‐test, ^***^ *p* < 0.001, error bars represent the SEM.

To further investigate the function of PTPN14 in breast cancer, stable PTPN14‐HA transfectants were generated in MDA‐MB‐231 and BT549 cells (**Figure** **3**A; Figure S3A, Supporting Information). In contrast to the effect of PTPN14 depletion, PTPN14‐overexpressing (PTPN14‐OE) breast cancer cells displayed diminished proliferation when cultured in monolayers, as evidenced by the results of the CCK‐8 and colony formation assays (Figure 3B; Figure S3B,D, Supporting Information). When these cells were cultured under ULA conditions, the PTPN14‐OE group exhibited reduced cell viability compared to the control group (Figure 3C; Figure S3C, Supporting Information). To determine whether the decline in the viable cell count among PTPN14‐OE cells under ULA conditions was attributable to anoikis, we assessed the apoptotic status of PTPN14‐OE and control cells under ULA culture conditions using Western Blotting and flow cytometry. The findings indicated that, in comparison to the control group, the PTPN14‐OE group exhibited an increase in caspase‐3 cleavage and that of its substrate PARP, along with a higher percentage of apoptotic cells in the total cell population (Figure 3D; Figure S3E, Supporting Information).

After culturing the cells under ULA conditions for 12 h, the phosphorylation levels of AKT Ser473 and ERK1/2 in PTPN14‐OE breast cancer cells were lower than those of the control group cells (Figure 3E; Figure S3F, Supporting Information). Consistent with the results of PTPN14 knockout, PTPN14‐OE cells exhibited lower phosphorylation levels of AKT Ser473 under EGF stimulation compared to control cells. Additionally, no significant difference in ERK1/2 phosphorylation levels was observed between the two groups (Figure 3F).

In the 3D invasion assays, compared to PTPN14 knockout, PTPN14 overexpression has the opposite effect, which is a significant reduction in the number of invasive clonal spheroids (Figure 3G; Figure S3G, Supporting Information). We also examined the alterations in tumor growth following PTPN14 overexpression. At different time points postinjection, the measurement data of tumor size showed that the tumor growth rate in the control group was faster than that in the PTPN14‐OE group (Figure 3H). Two groups of tumor weight also reflected this result (Figure 3I). There was no significant difference in body weight between the two groups of mice (Figure S3H, Supporting Information). Histological examination of the tumor sections showed that PTPN14‐OE group tumors exhibited no evidence of tissue necrosis and a lower number of tumor cells, accompanied by abundant residual Matrigel. Conversely, tumors in the control group displayed significant necrotic areas (dashed line annotation), indicating that PTPN14 overexpression substantially suppressed tumor growth. CD31 staining results demonstrated a decrease in microvascular structure formation in the PTPN14‐OE group tumors, in contrast to the control group. There was also a higher proportion of cells expressing the proliferation marker Ki‐67 in the control group compared to that of the PTPN14‐OE group (Figure 3J; Figure S3I, Supporting Information).

To further validate the impact of PTPN14 on the metastatic properties of breast cancer cells, particularly on anoikis resistance, we injected MDA‐MB‐231‐Luc (luciferase) cells overexpressing PTPN14 and their control counterparts into NOD SCID mice via the tail vein (Figure S3J, Supporting Information). The inhibition of lung metastasis in vivo was assessed using the bioluminescence imaging system. As depicted in Figure 3K and Figure S3K (Supporting Information), on the 41st‐day postinjection, ex vivo lung imaging showed significantly lower bioluminescent signals in the PTPN14 overexpression group compared to the control. This finding was supported by H&E staining and quantification of tumor metastasis locus number as shown in Figure 3L. Taken together, the experimental metastasis model indicated the inhibitory role of PTPN14 in cancer invasiveness.

These results collectively substantiate that PTPN14 overexpression inhibits anoikis resistance and in vivo tumorigenicity and pulmonary metastasis in TNBC cells.

### 2.4 Immunoprecipitation Followed by Mass Spectrometry (IP‑MS) Indicated That PTPN14 Interacted with BCAR3

**Figure 4:**
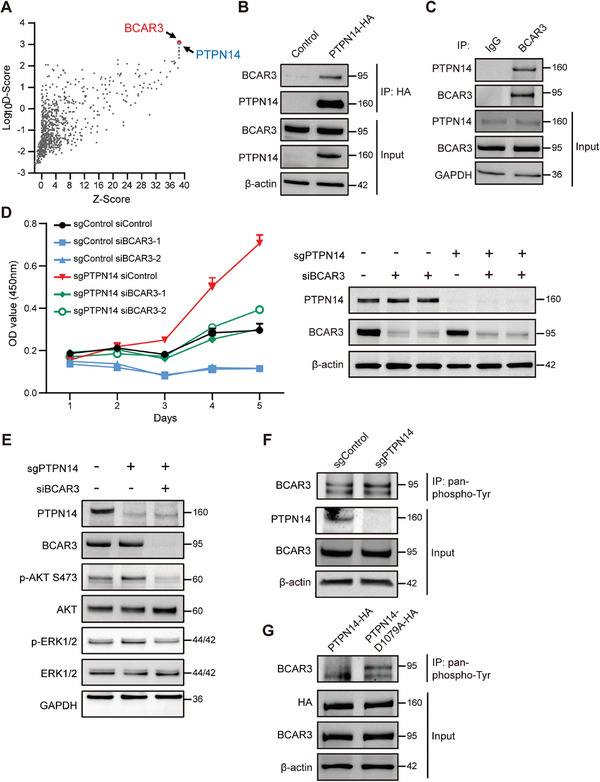
BCAR3 was identified as a substrate of PTPN14. A) The results of IP‐MS, following *CompPASS* analysis, yielded high‐confidence candidate PTPN14‐interacting proteins. B) Exogenous IP‐Western Blot analysis validated the interaction of PTPN14 and BCAR3. Three independent experiments were performed. C) Endogenous IP‐Western Blot analysis validated the interaction of PTPN14 and BCAR3. D) BCAR3 was knockdown in control or PTPN14‐KO MDA‐MB‐231 cells and cell viability under ULA conditions was assessed every 24 h for 5 days using the CCK8 assay (left); validation of protein expression levels in each cell group through Western Blotting (right). Three independent experiments were performed. E) After the knockdown of BCAR3 in PTPN14‐KO MDA‐MB‐231 cells, the phosphorylation levels of AKT and ERK were assessed. Three independent experiments were performed. F) Tyrosine phosphorylated protein IP‐Western Blot analysis was conducted in both control MDA‐MB‐231 cells and PTPN14‐KO MDA‐MB‐231 cells. Three independent experiments were performed. G) Tyrosine phosphorylated protein IP‐Western Blot analysis was conducted in both PTPN14‐HA MDA‐MB‐231 cells and PTPN14‐D1079A‐HA MDA‐MB‐231 cells.

To better understand the mechanism by which PTPN14 regulates anoikis resistance and tumorigenic potential in TNBC cells, we performed IP‐MS analysis to identify its substrates. Using C‐terminal HA‐tagged PTPN14 as “bait” overexpressed in MDA‐MB‐231 cells and employing dimethyl dithiobispropionimidate (DTBP) as a crosslinker to stabilize protein‐protein interactions, the IP‐MS data were scored by Comparative Proteomic Analysis Software Suite (*CompPASS*) to identify high‐confidence candidate PTPN14‐interacting proteins (**Figure** **4**A).^[^ ^18^ ^]^ The results were ranked by the two metrics of *CompPASS* (Z‐score and D‐score), which revealed that BCAR3 scored the highest. BCAR3 possesses multiple tyrosine phosphorylation sites,^[^ ^19^ ^]^ suggesting that BCAR3 may be a substrate for PTPN14.

We detected protein–protein interactions between PTPN14 and BCAR3 in MDA‐MB‐231 cells using exogenous and endogenous co‐immunoprecipitation (co‐IP) assays. Consistent with the IP‐MS results, the results of both exogenous and endogenous co‐IP confirmed their interactions (Figure 4B,C). To investigate whether PTPN14 functions in breast cancer through BCAR3, we used siRNA specifically targeting BCAR3 to downregulate its expression in PTPN14‐KO and control cells. Remarkably, the knockdown of BCAR3 reversed the growth advantage observed in PTPN14‐KO MDA‐MB‐231 cells under ULA conditions (Figure 4D). Furthermore, we noted sustained upregulation of BCAR3 expression within 3 days of ULA culture, underscoring the significant role of BCAR3 in mediating anoikis resistance in TNBC cells (Figure S4A, Supporting Information). To further investigate the connection between PTPN14 and BCAR3, we explored the possibility of reversing the activation of the PI3K/AKT and ERK signaling pathways induced by PTPN14 knockout through the knockdown of BCAR3. As illustrated in Figure 4E, increased phosphorylation of AKT Ser473 and ERK1/2, which was evident after PTPN14 knockout, was mitigated by BCAR3 knockdown.

To further investigate the role of BCAR3 in the regulation of AKT and ERK signaling pathways by PTPN14, we examined the phosphorylation levels of AKT and ERK following BCAR3 knockdown. The results from MDA‐MB‐231 and BT549 cells showed that phosphorylation levels of AKT Ser473 and ERK1/2 significantly decreased after BCAR3 knockdown, indicating that BCAR3 is at the upstream of these two signaling pathways and involved in their regulation (Figure S4B, Supporting Information).

To further investigate whether PTPN14 directly regulates BCAR3 phosphorylation, we performed immunoprecipitation (IP) experiments in both PTPN14‐KO and control MDA‐MB‐231 cells to enrich the tyrosine‐phosphorylated proteins. Subsequently, we probed BCAR3 within these proteins using BCAR3 antibodies in a Western Blot. The results showed that although the expression level of BCAR3 remained unchanged following the knockout of PTPN14, there was an increased pull‐down of BCAR3 through the enrichment of tyrosine‐phosphorylated proteins. This suggests an elevated phosphorylation level of BCAR3 following the knockout of PTPN14 (Figure 4F).

To demonstrate that BCAR3 is a substrate of PTPN14, we generated two stable MDA‐MB‐231 cell lines: one expressing wild‐type PTPN14 and the other expressing the PTPN14‐D1079A mutant. This mutant has been shown to trap phospho‐substrates within its catalytic pocket, safeguarding them from dephosphorylation, and potentially enhancing the enrichment of phosphorylated substrates.^[^ ^6^, ^20^ ^]^ Through IP experiments in these cell lines to enrich tyrosine‐phosphorylated proteins, we found that despite no change in BCAR3 expression, IP samples from the PTPN14‐D1079A mutation group exhibited a higher enrichment of BCAR3. This indicated that the substrate‐trapping mutant PTPN14‐D1079A enhanced the phosphorylation of BCAR3 (Figure 4G).

Collectively, these results provide evidence that BCAR3 is a phospho‐substrate for PTPN14 and that the function of PTPN14 in breast cancer anoikis and tumorigenicity involves BCAR3.

### 2.5 Synthesis and Expression of PTPN14 mRNA

**Figure 5:**
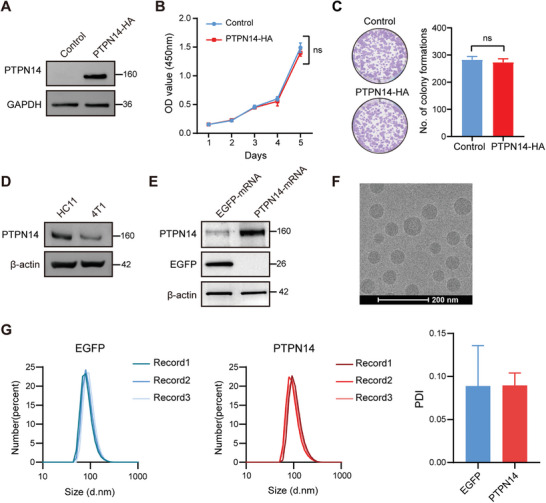
Expression of PTPN14 mRNA in vitro. A) Validation of PTPN14 overexpression in MCF10A cells through Western Blotting. Three independent experiments were performed. B) Cell proliferation of the control and PTPN14‐OE MCF10A cells in monolayer adherent culture were assessed every 24 h for 5 days using the CCK‐8 assay (*n* = 3). Student's *t*‐test, with *p* < 0.05 indicating statistical significance. C) Representative images of colony formation assays in the control and PTPN14‐OE MCF10A cells (left), along with statistical summaries of the results from three independent experiments (right). Student's *t*‐test, with *p* < 0.05 indicating statistical significance. D) Western Blot analysis compared the expression level of PTPN14 in HC11 cells and 4T1 cells. Three independent experiments were performed. E) After transfection of HEK293T cells with PTPN14 mRNA and EGFP mRNA separately for 24 h, the expression of PTPN14 was assessed by Western Blot. Three independent experiments were performed. F) Cryo‐TEM images of PTPN14 mRNA‐LNPs. G) Particle size distribution and PDI of LNPs. d.nm, diameter (nm).

To ensure that upregulation of PTPN14 expression did not adversely affect the surrounding normal tissue, we initially overexpressed PTPN14 in human mammary epithelial cells (MCF10A) (**Figure** **5**A). Contrary to the results observed in breast cancer cells, CCK‐8 and colony formation assays indicated that the overexpression of PTPN14 did not affect MCF10A cell proliferation (Figure 5B,C). We also compared the expression levels of PTPN14 in mouse mammary epithelial cells HC11 and mouse triple‐negative breast cancer cells 4T1. Western Blot analysis showed that PTPN14 expression was higher in HC11 cells than in 4T1 cells (Figure 5D). These findings support the feasibility of restoring PTPN14 expression in breast cancer. Utilizing a codon‐optimized mouse PTPN14 template, we synthesized chemically modified PTPN14 mRNA via IVT. After transfecting PTPN14 mRNA into HEK293T cells, we observed a significant increase in the expression level of PTPN14 within 24 h (Figure 5E).

Because of the inherent instability of mRNA in the circulatory system and its susceptibility to degradation by RNase, we employed a strategy involving encapsulation of mRNA in lipid nanoparticles (LNPs) to achieve efficient mRNA transfection in vivo.^[^ ^20^ ^]^ The cryo‐TEM images in Figure 5F validate the size of the LNPs and successful PTPN14 mRNA encapsulation. The mRNA‐LNPs had an ≈100 nm diameter and showed a polydispersity index (PDI) of 0.05–0.15, as measured with Malvern Zetasizer Nano ZS (Figure 5G).

### 2.6 Therapeutic Efficacy of Intratumoral Injections of PTPN14 mRNA in 4T1 Tumors

**Figure 6:**
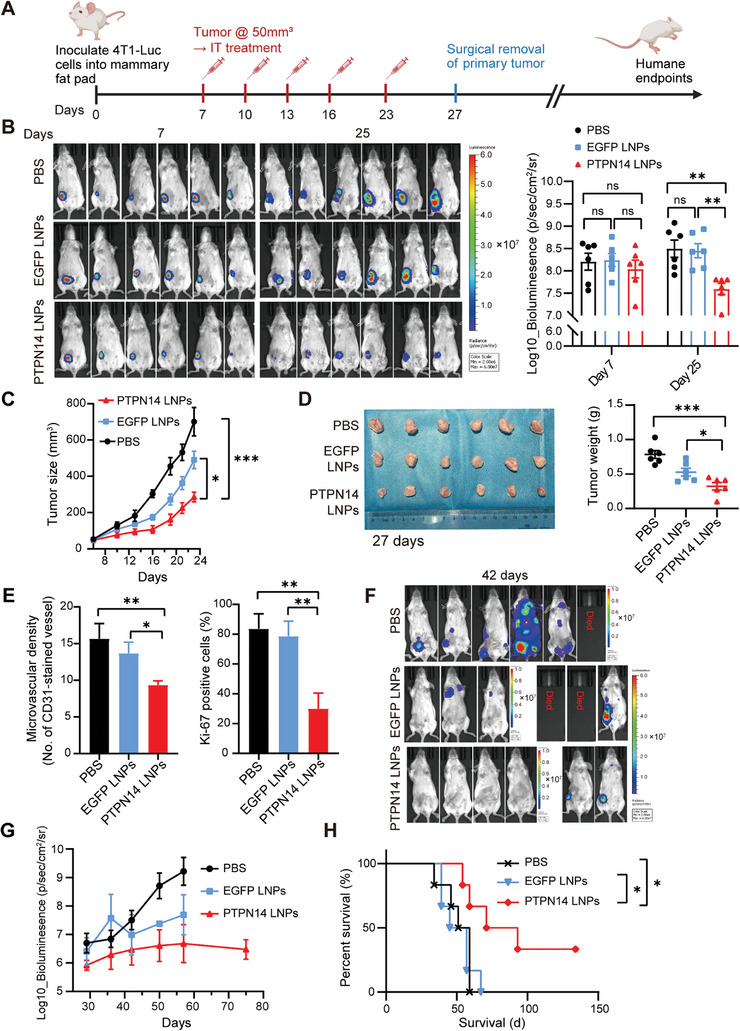
Effects of PTPN14 mRNA‐LNPs on the growth and metastasis of 4T1 tumors. A) The workflow diagram of a therapeutic experiment involving PTPN14 mRNA‐LNPs in the 4T1 tumor model. B) In vivo imaging of mice from each group on the 7th and 25th days post‐4T1 tumor inoculation (left), along with the corresponding quantification of bioluminescence intensity (right) (*n* = 6). Tukey's multiple comparisons test following one‐way ANOVA, ^**^ *p* < 0.01, error bars represent the SEM. C) The growth curves of tumors in response to different treatments (*n* = 6). Tukey's multiple comparisons test following one‐way ANOVA, ^*^ *p* < 0.05, ^***^ *p* < 0.001, error bars represent the SEM. D) On the 27th day post‐4T1 tumor inoculation, the orthotopic tumors were surgically excised, photographed (left), and weighed (right) (*n* = 6). Tukey's multiple comparisons test following one‐way ANOVA, ^*^ *p* < 0.05, ^***^ *p* < 0.001, error bars represent the SEM. E) Quantification of CD31 and Ki‐67 IHC staining results of three groups of tumor sections (*n* = 3). Tukey's multiple comparisons test following one‐way ANOVA, ^*^ *p* < 0.05, ^**^ *p* < 0.01. F) In vivo imaging of mice from each group on the 42nd day post‐4T1 tumor inoculation. G) Quantification of bioluminescence intensity of total tumor burden for each group from days 29–75 post‐4T1 tumor inoculation. Error bars represent the SEM. H) Survival curves. Log‐rank [Mantel–Cox] test, ^*^ *p* < 0.05.

We evaluated the effects of PTPN14 mRNA‐LNPs on orthotopic 4T1‐Luc tumors, which is one of the most extensively used models of TNBC. With high tumorigenicity and invasiveness, 4T1 tumors can spontaneously metastasize from primary mammary gland tumors to multiple distant sites, closely mirrors human TNBC.^[^ ^21^ ^]^ **Figure** **6**A shows the workflow of the experiments. Seven days after orthotopic tumor implantation, in vivo imaging showed no significant differences in the bioluminescent signals among the three groups of tumors. Given that primary breast tumor lesions are often located close to the body surface, we opted for intratumoral injections to circumvent potential liver accumulation associated with intravenous injections. After administration at 7, 10, 13, 16, and 23 days, in vivo, imaging showed that the primary tumors in the PTPN14 mRNA‐LNPs treatment group lost or weakened their bioluminescent signals and were significantly lower than those in the two control groups (PBS and EGFP mRNA‐LNPs treatment) on day 25 (Figure 6B). Corresponding to this result, tumor growth in the PTPN14 mRNA‐LNPs treatment group was significantly slower than that in the two control groups (Figure 6C). During the treatment period, no significant differences in body weight were observed between the three groups of mice (Figure S5A, Supporting Information). After 27 days, all mice were anesthetized and their orthotopic tumors were surgically removed. This is because 4T1 primary tumors reach the maximum allowable size in ≈30 days, at which point there is insufficient detectable metastatic disease. The premature death of mice due to the burden of primary tumors also prevented an objective comparison of their metastatic relapse status. Figure 6D shows that the primary tumors in the PTPN14 mRNA‐LNPs treatment group were notably smaller and lighter than those in the two control groups. The IHC results for CD31 and Ki‐67 also showed that the degree of vascularization and proliferation of tumors in the PTPN14 mRNA‐LNPs treatment group was reduced (Figure 6E; Figure S5B, Supporting Information).

Based on our previous experimental results (unpublished data), the half‐life of IVT‐mRNA expression in tumors is less than 24 h. As 96 h had passed between the final treatment and the surgical removal of the primary tumor, the PTPN14 expression level in IHC staining of the three groups of tumor tissues showed no significant differences (Figure S5C, Supporting Information).

These results indicated that intratumoral injections of PTPN14 mRNA‐LNPs could suppress the growth of TNBC tumors.

Furthermore, histological analysis of the tumor sections demonstrated that in the PBS and EGFP mRNA‐LNPs treatment groups, the tumor cells infiltrated the surrounding tissue and invaded the muscular layer. In contrast, the boundary between the tumor and normal tissues was well‐defined in the PTPN14 mRNA‐LNPs treatment group, indicating that therapy with PTPN14 mRNA‐LNPs reduced tumor progression (Figure S5B, Supporting Information).

In the PBS treatment group, imaging conducted on the 2nd day following the surgical removal of primary tumors already exhibited widespread metastasis, characterized by rapid progression that resulted in the first mortality within 34 days. Furthermore, more than two‐thirds of the mice experienced systemic metastasis before reaching the endpoint. In the two control groups, 50% of the mice experienced tumor relapse at the surgical site within 36 days and reached the humane endpoints specified in the Animal Ethics and Welfare Guidelines within 51 days, whereas the remaining 50% experienced metastatic relapse (Figure 6H; Figure S5D, Supporting Information). In contrast, the PTPN14 mRNA‐LNPs treatment group showed delayed and milder instances of relapse and metastasis. Quantification of bioluminescence intensity of total tumor burden for each group from days 29–75 post‐4T1 tumor inoculation is shown in Figure 6G. Taking imaging from 42 days as an example, within the PBS group, all surviving mice exhibited metastasis, whereas this proportion was 75% in the EGFP‐LNPs treatment group. Notably, there were no deaths in the PTPN14‐LNPs treatment group, and two‐thirds of the mice in this group showed no bioluminescent signals throughout their bodies (Figure 6F). After 75 days, when mice in both control groups reached the endpoint, all surviving mice in the PTPN14 mRNA‐LNPs treatment group showed no bioluminescent signals other than those at the primary site (Figure S5D, Supporting Information). The median survival times were 55 days (PBS), 51 days (EGFP‐LNPs), and 82 days (PTPN14‐LNPs) (Figure 6H). Autopsies of mice reaching the endpoint showed metastatic sites extending beyond the common locations in the lungs, liver, and axillary lymph nodes. These sites encompassed vascular‐rich and highly perfused areas such as the chest wall, mesentery, and thyroid gland. Additionally, we observed significant enlargement of the axillary lymph nodes with evidence of fusion (Figure S5E, Supporting Information).

These findings demonstrate that intratumoral injection of PTPN14 mRNA can inhibit the growth of primary 4T1 tumors, delay and reduce the occurrence of metastatic relapse, markedly improve prognosis, and significantly prolong the survival of animals.

## 3 Discussion

It is unclear how disseminated tumor cells (DTCs) gain the ability to survive without anchorage, particularly in TNBC. In this study, using a genome‐wide CRISPR/Cas9 knockout screen, we discovered that PTPN14 knockout TNBC cells exhibit a significant survival advantage under detached conditions. We further elucidated that by dephosphorylating BCAR3, PTPN14 downregulated the activation of the ERK and PI3K/AKT signaling pathways, consequently leading to the induction of anoikis. The intratumoral injection of LNPs‐encapsulated PTPN14 mRNA successfully inhibited the growth of primary tumors, delayed and reduced the occurrence of distant metastasis, retarded tumor progression, and extended survival.

PTPN14 functions as a component of numerous signaling cascades that are either dependent or independent of its enzymatic activity. Our study elucidated PTPN14's role in enhancing the sensitivity of TNBC cells to anoikis and suppressing their tumorigenicity in an enzyme activity‐dependent manner. In line with this finding, a recent large‐scale genome‐wide association study has indicated that functional loss variants of PTPN14 confer a significant risk for basal cell carcinoma.^[^ ^7b^ ^]^

Importantly, we identified a novel protein interacting with PTPN14, BCAR3 through IP‐MS and confirmed PTPN14's dephosphorylation of BCAR3. As a member of the NSP (novel SH2‐containing protein) family, BCAR3 forms complexes with breast cancer antiestrogen resistance protein 1 (BCAR1). Serving as a crucial downstream adaptor in integrin and growth factor receptor signaling, BCAR1 undergoes phosphorylation upon receiving signals from FAK and Src. This initiates the recruitment of the corresponding adaptor proteins, ultimately activating pro‐survival signaling pathways, including ERK and PI3K/AKT.^[^ ^22^ ^]^ In fact, the tyrosine phosphorylation of BCAR3 enhances integrin‐induced BCAR1 tyrosine phosphorylation,^[^ ^23^ ^]^ which, in turn, activates BCAR1 and its downstream signaling.^[^ ^24^ ^]^ A prior study noted PTPN14's role in regulating BCAR1 phosphorylation,^[^ ^25^ ^]^ and our study identified BCAR3 as a PTPN14 substrate. We hypothesized that PTPN14‐mediated dephosphorylation of BCAR3 leads to reduced BCAR3 phosphorylation, thereby inhibiting the phosphorylation and activation of BCAR1 and subsequently suppressing pro‐survival signal transmission. Further investigations are needed to explore whether the dephosphorylation of BCAR3 by PTPN14 affects its interaction with BCAR1 and the related pathways.

Overactivation of the PI3K/AKT and ERK pathways not only confers anoikis resistance but also drives tumor initiation and progression. Notably, the upstream regulatory factors of these pathways, as well as frequent amplifications or activating mutations in AKT, are present in a variety of solid tumors. Furthermore, the AKT and ERK signaling pathways downstream consist of a series of pro‐survival and pro‐growth effectors that contribute to their tumorigenic effects.^[^ ^16^, ^25^, ^26^ ^]^ In normal epithelial cells, excessive activation of pro‐survival signaling pathways is absent.^[^ ^4^ ^]^ Therefore, overexpressing PTPN14, which negatively regulates the PI3K/AKT and ERK pathways, did not result in observable changes in cell proliferation in normal epithelial cells. Recently, the development of inhibitors targeting AKT and ERK has garnered widespread attention. Several AKT inhibitors are currently in phase III clinical trials,^[^ ^27^ ^]^ while the ERK inhibitor TIC10 has received FDA approval for the treatment of H3K27M‐mutated glioma.^[^ ^28^ ^]^ The discovery that PTPN14 inhibits the phosphorylation and activation of AKT and ERK highlights the potential of PTPN14 as a target for cancer therapy.

Due to the lack of therapeutic targets, patients with TNBC cannot benefit from molecular‐targeted therapies (such as endocrine therapy and anti‐HER2 therapy). Additionally, the extensive heterogeneity makes TNBC highly prone to developing resistance to chemotherapy, rendering its therapeutic management highly challenging.^[^ ^29^ ^]^ With significant breakthroughs in the treatment of TNBC using immunotherapy, some immune checkpoint blockers combined with other drugs have received FDA approval for TNBC treatment. However, the therapeutic scope in clinical practice remains limited.^[^ ^30^ ^]^ Therefore, there is an urgent need to explore new strategies for functional drug development for TNBC.^[^ ^31^ ^]^

Synthetic mRNA has demonstrated great potential in biomedical applications, including vaccine development, protein replacement, and gene editing. Notably, recent achievements in the systemic delivery of tumor suppressor mRNAs, such as PTEN and p53,^[^ ^13a,b^ ^]^ to tumor models have shown substantial inhibition of tumor growth by reinstating the expression of these suppressors through mRNA LNPs. In our study, the restoration of PTPN14 expression in primary tumor tissue not only inhibited tumor growth, suggesting a potential reduction in the number of tumor cells disseminated into the bloodstream, but also induced anoikis. This made it more challenging for DTCs with restored PTPN14 expression to survive and colonize secondary sites when detached. Even without systemic administration of PTPN14 mRNA, mice treated with PTPN14 mRNA, compared to the control group, showed alleviated tumor metastasis, suppressed cancer progression, and achieved complete remission in one case within the PTPN14 mRNA LNPs treatment group. However, our PTPN14 mRNA therapy has limitations. Intratumoral injection of PTPN14 mRNA LNPs did not restore PTPN14 protein expression in DTCs that had entered the bloodstream prior to treatment. Additionally, the transient nature of mRNA expression implies the need for multiple administrations.

With the continuous optimization of IVT of mRNA and the ongoing advancements in delivery platforms, the field of mRNA protein replacement therapy is accelerating. Achieving cancer cell‐specific delivery will enable PTPN14 mRNA to target tumor cells effectively, positioning it as a powerful anticancer drug. Restoration of PTPN14 represents a new approach to PI3K‐AKT pathway inhibition, with the potential to significantly improve the treatment of TNBC patients.

## 4 Conclusion

In conclusion, our data provide new insights into the anticancer effects of PTPN14, particularly in the processes of anoikis. Furthermore, mRNA therapy aimed at restoring PTPN14 inhibits both tumor growth and metastasis. These promising results may pave the way for the translational application of PTPN14 in cancer therapy, potentially extending the life expectancy of TNBC patients who have limited treatment options.

## 5 Experimental Section

### Cell Culture and Reagents

Human TNBC (MDA‐MB‐231 and BT549), human mammary epithelial (MCF10A), mouse TNBC (4T1), and mouse mammary epithelial cell lines (HC11) were obtained from the Cell Bank of the Chinese Academy of Sciences (Shanghai, China). Additionally, the human embryonic kidney cell line (HEK293T) was acquired from the American Type Culture Collection. BT549 and HEK293T cells were cultured in high‐glucose Dulbecco's modified Eagle's medium (DMEM; L110KJ, Basal media, Shanghai, China) supplemented with 20% and 10% fetal bovine serum (FBS; 40130ES76, Yeasen, Shanghai, China), respectively. Other cells were cultured in Roswell Park Memorial Institute (RPMI)−1640 medium (L210KJ, Basalmedia, Shanghai, China) supplemented with 10% FBS (40130ES76; Yeasen, Shanghai, China). All media contained 1% penicillin–streptomycin (S110JV; Basalmedia, Shanghai, China), and cells were grown in a humidified incubator at 37 °C with 5% CO2. In the EGF stimulation experiment, EGF (HY‐P7109, MedChemExpress, USA) was used to treat the cells. In the experiments assessing the effects of AKT inhibitor and ERK inhibitor on anoikis resistance, MK‐2206 (A3010, ApexBio, USA) and GSK1120212 (A3018, ApexBio, USA) were used to treat the cells.

All cell lines underwent STR profiling and regular testing for mycoplasma contamination using the Mycoplasma PCR Detection Kit (C0301S; Beyotime, Shanghai, China) and DAPI staining to ensure authenticity and absence of mycoplasma.

### Genome‑Wide CRISPR/Cas9 Screen

We obtained a Human GeCKOv2 CRISPR knockout pooled library from Feng Zhang (Addgene #1000000048).^[^ ^32^ ^]^ This lentiviral library was used to infect 5 × 107 MDA‐MB‐231 cells at a multiplicity of infection (MOI) of 0.3 to ensure single‐virus entry per cell. After puromycin screening, the MDA‐MB‐231 GeCKO cells were generated and divided into two groups. One group was cultured on regular plates, whereas the other was cultured on ULA plates (3471; Corning, USA). After 7 days, genomic DNA was extracted from ≈5 × 107 cells in each group using a Quick‐DNA Midiprep Plus Kit (D4075, Zymo Research, USA). Genomic DNA from both groups was subjected to PCR amplification of sgRNA fragments, purification of PCR products, and next‐generation sequencing (NGS) with each group yielding 50 million reads. The data was analyzed using MAGeCK and ranked genes based on RRA scores and *p* values.^[^ ^33^ ^]^

### Data Sources and DEGs Analysis

RNA sequencing data and the corresponding clinical information were obtained for 113 breast cancer‐adjacent tissues and 115 triple‐negative breast cancers from TCGA database. The dataset GSE191230 was also downloaded from the Gene Expression Omnibus (GEO) database, which includes RNA sequencing data for 13 primary breast tumors and seven distant metastatic tumors. Differential expression analysis was performed separately for the TCGA‐TNBC and GSE191230 cohorts using the R package “DESeq2.” The DEGs from TCGA‐TNBC and GSE191230 cohorts were then intersected with the positive genes from the CRISPR‐based screen, and a Venn diagram was generated using the online tool Hiplot (https://hiplot.com.cn/). The R package “ggpubr” was used to create box plots comparing the gene expressions of *PTPN14*, *APOLD1*, *FGL2*, and *LDLRAD2*. Additionally, the R package “ggplot2” was used to create scatter plots comparing the MAGeCK Rank, RRA scores, *p* values, and log2FC values of *PTPN14*, *APOLD1*, *FGL2*, and *LDLRAD2*.

### Immunohistochemistry

Tissues were fixed in 4% paraformaldehyde and subsequently underwent tissue dehydration, paraffin embedding, and immunohistochemical staining procedures as described earlier.^[^ ^34^ ^]^ Paraffin sections of primary and metastatic breast cancer from 53 patients, along with normal breast tissue sections from 11 patients with macromastia, were obtained from the Department of Pathology, Xiangya Hospital, Central South University. PTPN14 (GB114467‐100; Servicebio, 1:1000) was used to detect the PTPN14 protein level in patient samples. For mouse tissues, immunohistochemical staining was performed using CD31 (28083‐1‐AP; Proteintech, 1:1000), Ki‐67 (12202S; Cell Signaling Technology, 1:200) and PTPN14 (GB114467‐100; Servicebio, 1:1000). Subsequent incubation was carried out using corresponding secondary antibodies, including HRP‐conjugated goat antimouse IgG (GB23301; Servicebio, 1:200) and HRP‐conjugated goat antirabbit IgG (GB23303; Servicebio, 1:200). Incubation with primary antibodies occurred overnight at 4 °C, while secondary antibodies were incubated at room temperature for 50 min. A DAB chromogenic kit (G1212; Servicebio) was used for DAB staining. Subsequently, hematoxylin (G1004, G1039, G1040; Servicebio) was employed for nuclear staining.

The CD31 immunohistochemical score was used to evaluate microvascular formation within tissues. First, areas were located with dense CD31‐positive cells in the 40× field of view, and then selected five nonoverlapping fields under the 200× field of view were to count the total number of microvessels.

The statistical method for analyzing Ki‐67 immunohistochemical results was to calculate the percentage of Ki‐67‐positive cells. Specifically, three interpretation areas were initially located in the 40× field of view, selected three nonoverlapping visual fields under the 200× field of view, and calculated the percentage of Ki‐67‐positive nuclei among the total nuclei.

Other immunohistochemical staining results were analyzed using the following methods: The intensity was scored as follows: 0, negative; 1, weak; 2, moderate; and 3, strong. The frequency of positive cells was defined as follows: 0, less than 5%; 1, 5% to 25%; 2, 26% to 50%; 3, 51% to 75%; and 4, greater than 75%. The final grade of staining was determined by multiplying the score of intensity with the score of frequency (values, 0–12).

### Gene Silencing

One day in advance, cells were seeded into six‐well plates and cultured until the cell density reached ≈50%. Transfection of 100 nm siNC (5′‐UUCUCCGAACGAGUCACGU‐3′), siBCAR3‐1 (5′‐CCGAGCGGCCACUCUGAGUAA‐3′) or siBCAR3‐2 (5′‐GCCCAACGAGUUUGAGUCAAA‐3′) was performed using Lipofectamine 2000 (11668019, Thermo Fisher Scientific, USA). After 72 h of transfection, the proteins were extracted for Western Blot analysis.

### Generation of PTPN14 knockout cells

sgRNA‐PTPN14‐1 (5′‐CGTTGTAGCGCCGTGTCCGG‐3′) and sgRNA‐PTPN14‐2 (5′‐TGTTATCGAGTGCACGCTGT‐3′) were designed using the online platform Benchling (https://www.benchling.com) and cloned into the lentiCRISPRv2 vector. Lentiviral infection was performed, and stable PTPN14 knockout cell lines were obtained after selection with the appropriate concentration of puromycin (B7587, ApexBio, USA) for 3–5 days.

### Plasmids Construction and Establishment of Stable Cell Lines

The PTPN14 plasmid was obtained from the DNA library of the College of Basic Medical Sciences, Shanghai Jiao Tong University. PTPN14 was cloned into the pHAGE‐HA‐Puro lentivirus vector to generate a C‐terminal HA‐tagged wild‐type PTPN14 overexpression plasmid. Subsequently, site‐directed mutagenesis was performed using primers 5′‐AATATACTGACTGGCCAGCTCACGGCTGTCCAGAAGA‐3′ and 5′‐TCTTCTGGACAGCCGTGAGCTGGCCAGTCAGTATATT‐3′ to construct the PTPN14 D1079A mutant overexpression plasmid. HEK293T cells were transfected with a lentiviral expression vector and packaging plasmids (VSV‐G, MPMG, Rev, and Tet2) using polyethyleneimine (Polyscience, USA). After 48 h, the supernatant containing lentiviral particles was collected and added to newly plated cells along with polybrene (8 µg mL^−1^) to enhance infection efficiency. After 48 h, puromycin (B7587; ApexBio, USA) was added at the appropriate concentration for selection and stable cell lines were obtained after 3–5 days.

### Western Blot Analysis

Cells were lysed on ice for 30 min using mammalian cell lysis buffer (50 mm Tris pH 7.5, 150 mm NaCl, 0.5% IGEPAL CA‐630 (I3021, Sigma‐Aldrich, USA)) containing EDTA‐free protease inhibitor (04693132001, Roche, Switzerland), phosphatase inhibitor (4906845001, Roche, Switzerland), and 1 mm phenylmethylsulfonyl fluoride (PMSF; 329‐98‐6, Amresco, USA). After lysing, the cell lysate was centrifuged at 13 000 × g for 20 min at 4 °C, and the protein concentration was determined using the Bradford assay. Equal amounts of protein were subjected to SDS‐PAGE gels for electrophoretic separation. Subsequently, the proteins were transferred onto nitrocellulose membranes, and the membranes were blocked with 5% skim milk at room temperature for 1 h. After washing with Tris‐Buffered Saline with Tween (TBST), the membranes were incubated with the primary antibodies overnight. On the following day, the membranes were incubated at room temperature with horseradish peroxidase (HRP)‐conjugated secondary antibodies for 1 h and then detected by enhanced chemiluminescence using the ChampChemi imaging system (Sage Creation Science).

The following antibodies were used for Western Blot analysis: Cell Signaling Technology: PTPN14 (13808S, 1:1000 dilution), Cleaved PARP (5625S, 1:1000 dilution), Cleaved Caspase‐3 (9661S, 1:1000 dilution), p‐AKT (Ser473) (4060S, 1:2000 dilution), AKT (pan) (4691S, 1:1000 dilution), p‐ERK1/2 (4370S, 1:2000 dilution), ERK1/2 (4695S, 1:1000 dilution), BCAR3 (24032S, 1:1000 dilution), and HA‐Tag (3724S, 1:1000 dilution); Proteintech: GAPDH (60004‐1‐Ig, 1:50000 dilution), β‐actin (66009‐1‐Ig, 1:20000 dilution) and GFP tag (50430‐2‐AP, 1:1000 dilution); ABclonal: active + pro Caspase‐3 (A19654, 1:1000 dilution).

### Cell Proliferation Assay

CCK‐8 assay was used to evaluate cell proliferation. The control and experimental groups were seeded in 96‐well plates. At specific time points, a CCK‐8 assay was conducted according to the manufacturer's instructions (K1018, ApexBio, USA). Cell proliferation was quantified by measuring the optical density at 450 nm (OD450) using a microplate reader (Tecan Infinite 200 PRO).

### Spheroid Formation Assay

Equal numbers of cells from the control and experimental groups were seeded in six‐well ULA plates (3471, Corning, USA). The culture medium was replenished every 3 days. After culturing under ULA conditions for 7 days, the number of living cells was determined using Trypan Blue staining.

### 3D Invasion Assay

The experimental methods were based on previously published articles.^[^ ^35^ ^]^ Specifically, the procedure was as follows: Using precooled tips, 100 µL of Matrigel (356234, Corning, USA) was aspirated and dispensed onto the bottom of precooled wells of a 24‐well plate. The Matrigel was allowed to solidify by incubating at 37 °C for 60 min. MDA‐MB‐231 cells were collected in RPMI‐1640 complete medium at a concentration of 5000 cells per mL, ensuring the formation of a single‐cell suspension by thorough pipetting. Then, 200 µL of the cell suspension was added to each well of the Matrigel‐coated 24‐well plate, resulting in 1000 MDA‐MB‐231 cells per well, and incubated at 37 °C for 60 min. A mixture of chilled RPMI‐1640 complete medium with Matrigel to a final concentration of 10% Matrigel was prepared, and mixed thoroughly, and 200 µL of the Matrigel/medium mixture was gently added to each well of the 24‐well plate. The cells were cultivated to the appropriate time points, observed, and photographed under a microscope. Every three days, 200–400 µL of fresh medium was replaced. On the 6th day of culture, random images of five fields per well were taken using a 5× objective lens. According to previously described statistical methods,^[^ ^35a^ ^]^ the clonal spheroids were classified into two categories: those with distinct protrusions as invasive clonal spheroids, and the others as noninvasive. The number of each type of clonal spheroids was counted, and the percentages were calculated.

### IP‑MS

IP‐MS was performed following established protocols.^[^ ^18^ ^]^ HA‐tagged PTPN14 stable MDA‐MB‐231 cells were collected from six fully grown 10‐cm tissue culture dishes and treated with 50 mM DTBP (20665, Thermo Fisher Scientific, USA) for crosslinking. After 15 min of rotation, crosslinking was stopped with 125 mm glycine. Cells were collected, washed three times with PBS, and subjected to protein extraction. The 10 mg of total protein was then incubated with α‐HA magnetic beads (88836, Thermo Fisher Scientific, USA) at 4 °C overnight with gentle inversion. Beads were washed with lysis buffer, and elution was performed using 0.1 m glycine (pH 2.0). The eluted samples were de‐crosslinked by shaking at 37 °C with 150 mM DTT (10197777001; Roche, Switzerland). Two‐thirds of the eluted samples were processed and analyzed using an LTQ Orbitrap Fusion Mass Spectrometer (Thermo Fisher Scientific, USA) coupled with an EASY‐nLC 1000 Liquid Chromatograph (Thermo Fisher Scientific, USA).

The original mass spectrometric data were analyzed and identified using PEAKS Studio based on the UniProt database (20180524, 20349). Subsequently, *CompPASS* was employed for scoring, and the identified candidate interacting proteins were ranked using the two metrics of *CompPASS* (Z‐Score and D‐Score).^[^ ^18^ ^]^

### Endogenous Immunoprecipitation

In accordance with the experimental requirements, cells were collected from 3 to 4 fully grown 10‐cm tissue culture dishes, and protein extraction was performed after crosslinking. To preclear the lysate, equal amounts of protein and Protein A/G PLUS‐Agarose (sc‐2003, Santa Cruz Biotechnology) were incubated at 4 °C with rotation for 2 h. The mixture was then centrifuged to remove the precipitate while retaining the supernatant. The corresponding antibody was added to the supernatant and incubated at 4 °C with rotation overnight. On the following day, Protein A/G PLUS‐Agarose was added to the supernatant and rotated at 4 °C for 3 h. After centrifugation, the supernatant was removed, and the pellet was retained. The obtained pellet was washed and de‐crosslinked as described above. A 5× SDS loading buffer was added and the mixture was boiled at 98 °C for 10 min. The resulting samples were used for Western Blot analysis.

The following antibodies were used for immunoprecipitation: BCAR3 (24032S, Cell Signaling Technology), antiphospho‐tyrosine (P‐Tyr‐100) (9411S, Cell Signaling Technology), and Rabbit IgG (A7016, Beyotime).

### Preparation of Modified PTPN14 mRNA

PTPN14 mRNA was synthesized in vitro through T7 RNA polymerase‐mediated RNA transcription, following established procedures.^[^ ^13^, ^36^ ^]^ The murine PTPN14 amino acid sequence was codon‐optimized by General Biology (Anhui, China), synthesized, and then cloned into the pcDNA3.1‐vector downstream of the T7 promoter. A poly(A)tail was introduced by PCR amplification of the DNA template, which was purified for IVT. In the IVT reaction, a Cap1 analog (EzCap AG; B8176, ApexBio, USA) was added to place a Cap1 structure at the 5′ end of mRNAs. Additionally, modified uridine analogs, specifically N1‐methyl‐pseudouridine (m1ψ), were used to fully replace uridine (U). DNase (EN0521, Thermo Fisher Scientific, USA) digestion was used to remove the DNA template, and the mRNA was subsequently purified using RNA Clean & ConcentratorTM‐25 (Zymo Research, USA).

### Preparation of mRNA‐Loaded Lipid Nanoparticles

The LNPs were prepared following established procedures.^[^ ^36^ ^]^ Lipids with a molar ratio of 50:10:38.5:1.5 (SM102: DSPC: cholesterol: PEG2000‐DMG) were dissolved in ethanol. They were then mixed with an equal volume of mRNA solution in 50 mm citrate buffer (pH 3.0) at a flow rate of 1 mL s^−1^. The resulting formulations were diluted twofold with 50 mm citrate buffer (pH 3.0) and dialyzed in PBS (pH 7.4) for a minimum of 15 h using dialysis cassettes. The resulting product was concentrated using Amicon Ultra Centrifugal Filters (MilliporeSigma, USA) and filtered through a 0.22‐µm filter. Particle size and PDI were assessed using a nanoparticle tracking analysis instrument (Zetasizer, Malvern PANalytical, USA).

### Cryo‐Transmission Electron Microscopy (cryo‐TEM) Sample Preparation and Imaging

A glow‐discharged grid (R2/1 Cu, 300 mesh, Quantifoil, Germany) was used to support the concentrated mRNA‐LNP samples. A Vitrobot Mark IV System (Thermo Fisher Scientific, USA) was used for cryofixation. Images were captured using a transmission electron microscope (Talos F200C G2, Thermo Fisher Scientific, USA) with the following settings: acceleration voltage, 200 kV; magnification, 36 000×; and pixel size, 5.75 Å.

### Animals Experiment

To establish a breast cancer xenograft orthotopic tumor model, 4–5 5‐week‐old female BALB/c nude mice were used. The mice were randomly divided into four groups of four mice each. Based on the grouping, MDA‐MB‐231‐Luc cells, MDA‐MB‐231‐PTPN14‐HA cells, MDA‐MB‐231‐sgControl cells, or MDA‐MB‐231‐sgPTPN14 cells were inoculated into the right fourth mammary fat pad of the mice, with each mouse receiving 8 × 105 cells. Tumor size and weight were regularly monitored and documented. At ≈6 or 9 weeks, the mice were humanely euthanized, and the tumors were excised, photographed, and weighed.

To establish a 4T1 breast cancer metastasis model, 6–7 weeks old female BALB/c mice were used. 4T1‐Luc cells were inoculated into the right fourth mammary fat pad of each mouse at a dose of 2.5 × 104 cells. Both the tumor size and mouse weight were regularly monitored and recorded. On the 7th day, when the tumors at the injection site reached ≈50 mm^3^ in size, the mice into three groups were randomly divided based on tumor size: the PBS treatment group, EGFP mRNA‐LNPs treatment group, and PTPN14 mRNA‐LNPs treatment group, with each group containing six mice. On days 7, 10, 13, 16, and 23, 50 µL of either PBS or LNPs (0.2 mg mL^−1^) was administered into the tumors of each group of mice. On the 27th day, the mice were anesthetized, surgically excised the tumors, and sutured the incisions. The physiological status and weight of the mice were regularly monitored, and those that reached the experimental endpoint were humanely euthanized. The dissected mice were photographed to observe tumor metastasis. The lungs were collected and stained with H&E. The mice were intraperitoneally injected with 150 mg kg^−1^ d‐luciferin (122799; PerkinElmer, MA, USA). Ten minutes after the luciferin injection, an IVIS Spectrum CT (128201, PerkinElmer, MA, USA) was used to monitor tumor growth and metastasis. The luminescence values were quantified using Living Image Software (PerkinElmer, MA, USA).

To establish an experimental breast cancer lung metastasis model, 6–7 weeks old female NOD SCID mice were used. MDA‐MB‐231‐Luc cells were infected with either pHAGE‐PTPN14‐HA lentivirus or its empty vector lentivirus to construct MDA‐MB‐231‐Luc cells stably overexpressing PTPN14 and their control cells. The NOD SCID mice were randomly divided into two groups, with six mice in each group. According to the group assignment, MDA‐MB‐231‐Luc cells overexpressing PTPN14, and the control cells were injected into the tail veins of NOD SCID mice at a dose of 1 × 106 cells in 100 µL PBS per mouse. On the 41st day after xenograft, an IVIS Spectrum CT was used to monitor tumor metastasis. Mice were sacrificed 10 min after luciferin injection, and the lungs were dissected within 5 min for ex vivo imaging, followed by weighing, fixation in 4% paraformaldehyde, paraffin embedding, H&E staining, and observation under a light microscope. Also, the luminescence values were quantified using Living Image Software.

### Ethics

In this study, BALB/c nude mice, BALB/c mice, and NOD SCID mice were used to establish breast cancer models. All animal experiments adhered to the Animal Ethics and Welfare Guidelines of the Chinese Ministry of Public Health and were approved by the Animal Care and Experiment Committee of Shanghai Jiaotong University School of Medicine with the accreditation number: 2019‐097‐01. Human pathological sections were obtained from the Pathology Department of Xiangya Hospital. Ethics approval for this study was granted by the Ethics Committee of Xiangya Hospital, Central South University with the project number: 202309143.

### Statistical Analysis

The densitometry values of the Western Blot bands were analyzed using ImageJ software. The normalized intensity of each target protein band was obtained by dividing the intensity of the target protein by the intensity of the corresponding loading control. The resulting normalized values were further processed by setting the control group as the reference. Unless stated otherwise, graphical data depict the mean ± SD from a minimum of three independent experiments. The statistical significance of survival curves was assessed using the log‐rank test. Comparisons between two groups of data were analyzed using a two‐tailed Student's *t*‐test, and multiple sets of data were analyzed with Tukey's multiple comparisons test following one‐way ANOVA or Dunn's multiple comparisons test following the Kruskal–Wallis test. *p* values less than 0.05 indicate statistical significance (ns, *p* > 0.05; ^*^ *p* < 0.05; ^**^ *p* < 0.01; and ^***^ *p* < 0.001). The data were analyzed and visualized using GraphPad Prism 8.0 (GraphPad Software Inc.).

## Conflict of Interest

The authors declare no conflict of interest.

## Supporting information

Supporting Information

Supplemental Table 1

## Data Availability

The data that support the findings of this study are available from the corresponding author upon reasonable request.
