# Supplementary material for: mRNA‐Lipid Nanoparticle‐Mediated Restoration of PTPN14 Exhibits Antitumor Effects by Overcoming Anoikis Resistance in Triple‐Negative Breast Cancer
Source: Adv Sci (Weinh). 2024 Jun 21;11(32):2309988. doi: 10.1002/advs.202309988 (PMC11348215; doi:10.1002/advs.202309988)
Supplement: Supplementary file 1 — Supporting Information [file ADVS-11-2309988-s002.pdf]

ADVANCED SCIENCE
Open Access

Supporting Information

for *Adv. Sci.*, DOI 10.1002/advs.202309988

mRNA-Lipid Nanoparticle-Mediated Restoration of PTPN14 Exhibits Antitumor Effects by Overcoming Anoikis Resistance in Triple-Negative Breast Cancer

Wei Li, Masha Huang, Zhaoping Wu, Yu Zhang, Ying Cai, Juncheng Su, Jia Xia, Fan Yang, Desheng Xiao, Wen Yang, Yingjie Xu* and Zhaoqian Liu**

Supporting Information

mRNA-Lipid Nanoparticle-Mediated Restoration of PTPN14 Exhibits Antitumor Effects by Overcoming Anoikis Resistance in Triple-Negative Breast Cancer

Wei Li[†]*, Masha Huang*[†]*, Zhaoping Wu, Yu Zhang, Ying Cai, Juncheng Su, Jia Xia, Fan Yang, Desheng Xiao, Wen Yang*, Yingjie Xu*, Zhaoqian Liu**

Figure S1

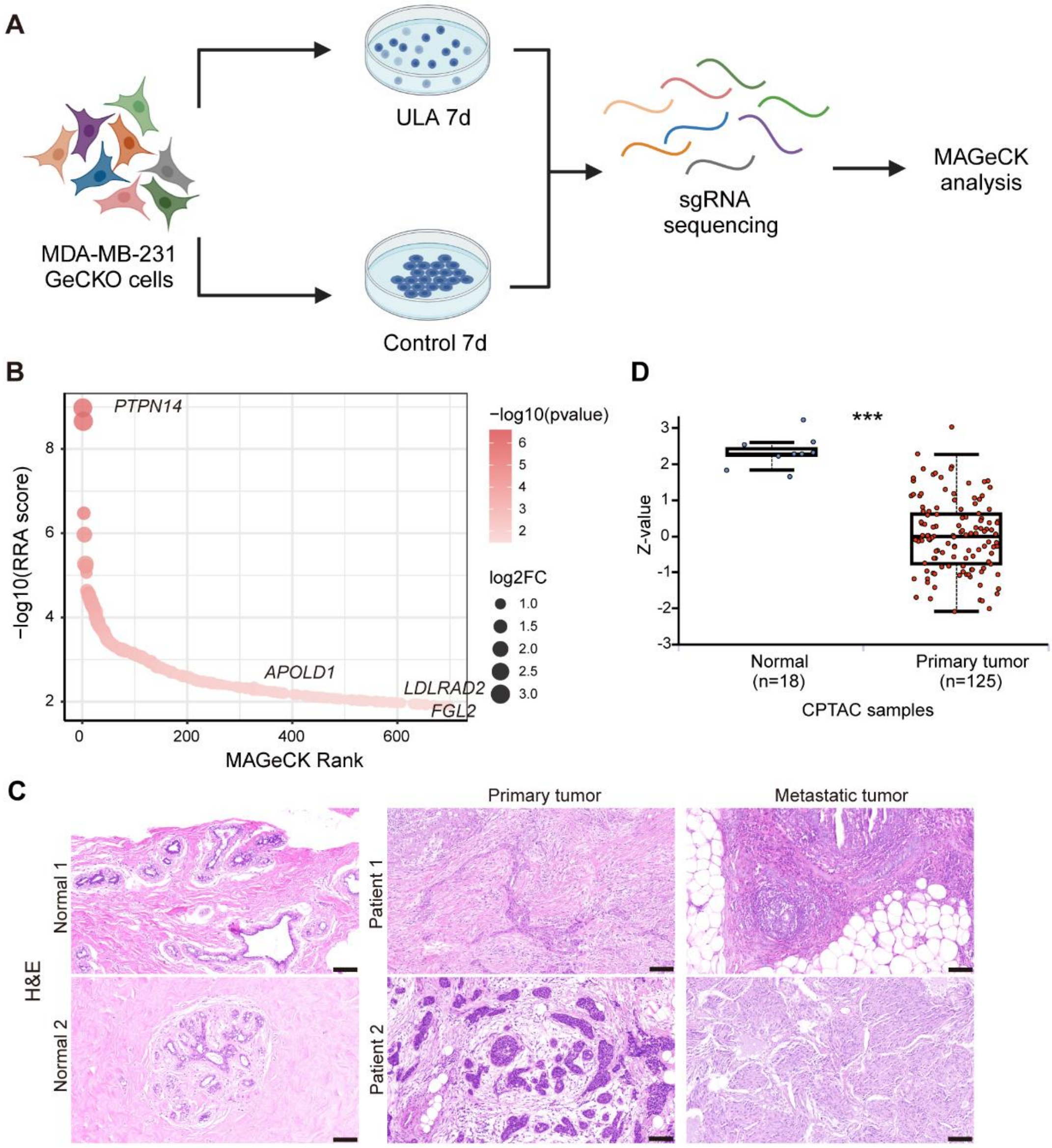

Figure S1. A pooled genome-wide CRISPR screen in a TNBC anoikis model.

(A) Schematic of CRISPR-based gene screening for anoikis resistance. (B) *PTPN14*, *APOLD1*, *FGL2*, and *LDLRAD2* ranked based on MAGeCK analysis results. (C) Representative H&E staining images of paired primary tumors and metastatic tumors (n=53), and normal breast tissue (n=11). Scale bar: 100 µm. (D) PTPN14 protein expression level in 18 normal breast samples and 125 primary breast tumor samples from CPTAC dataset. Student's t-test, ***p < 0.001.

Figure S2

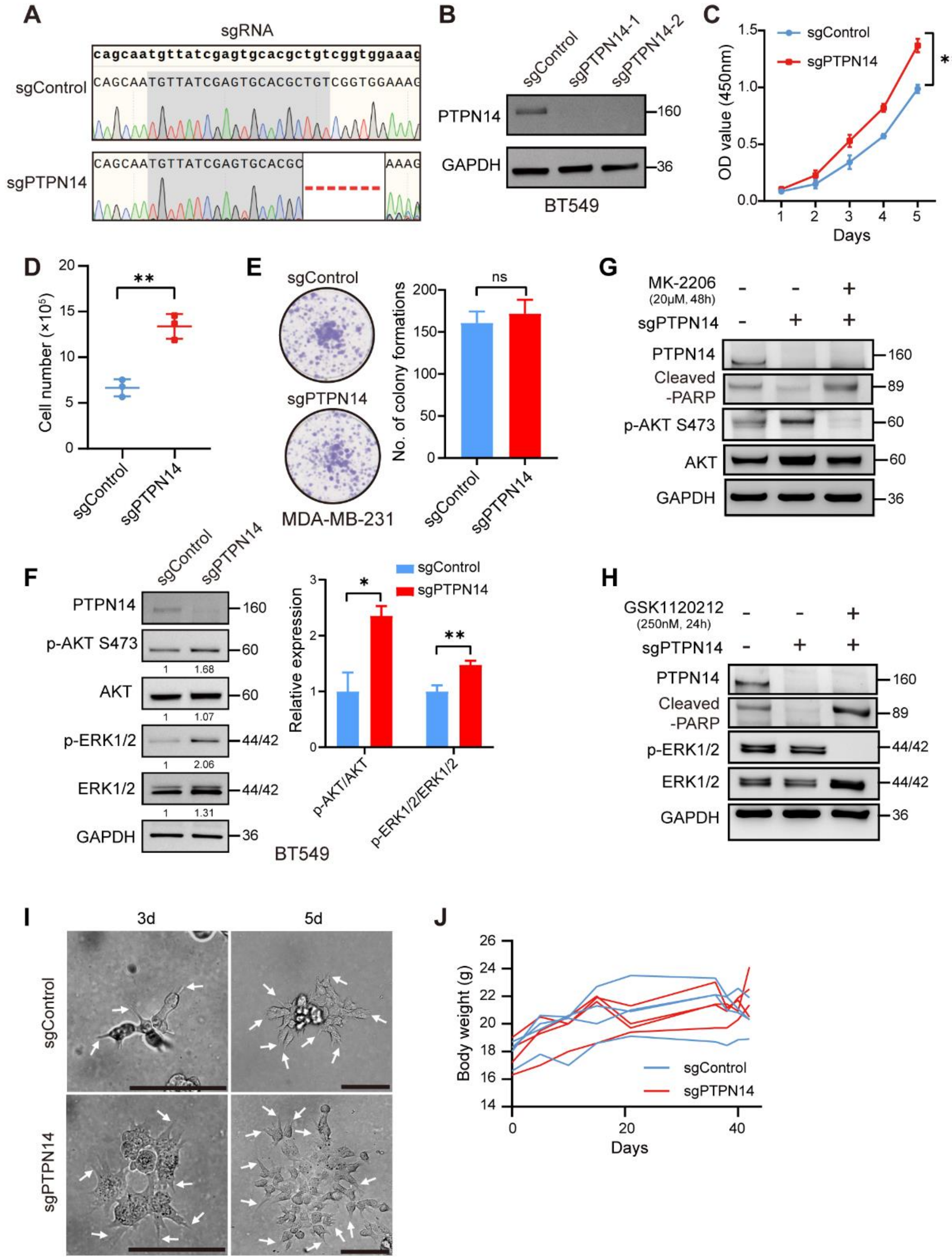
A
sgRNA
cagcaatgttatcgagtgcacgctgtcggtggaaag
sgControl
CAGCAATGTTATCGAGTGCACGCTGTCGGTGGAAAG
sgPTPN14
CAGCAATGTTATCGAGTGCACGC
AAAG
B
sgControl
sgPTPN14-1
sgPTPN14-2
PTPN14
GAPDH
160
36
BT549
C
sgControl
sgPTPN14
***
OD value (450nm)
Days
D
**
Cell number (×10^5)
sgControl
sgPTPN14
E
sgControl
sgPTPN14
MDA-MB-231
ns
No. of colony formations
G
MK-2206
(20μM, 48h)
sgPTPN14
PTPN14
Cleaved -PARP
p-AKT S473
AKT
GAPDH
160
89
60
60
36
F
sgControl
sgPTPN14
PTPN14
p-AKT S473
AKT
p-ERK1/2
ERK1/2
GAPDH
1 1.68
1 1.07
1 2.06
1 1.31
160
60
60
44/42
44/42
36
Relative expression
*
**
p-AKT/AKT
p-ERK1/2/ERK1/2
BT549
H
GSK1120212
(250nM, 24h)
sgPTPN14
PTPN14
Cleaved -PARP
p-ERK1/2
ERK1/2
GAPDH
160
89
44/42
44/42
36
I
3d
5d
sgControl
sgPTPN14
J
Body weight (g)
Days
sgControl
sgPTPN14

Figure S2-continued

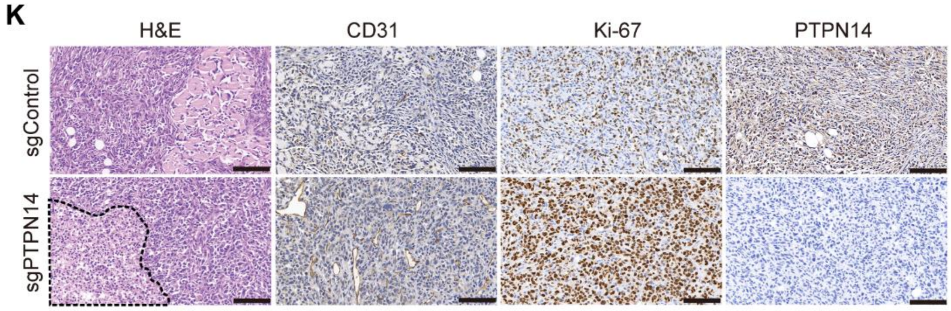

Figure S2. PTPN14 knockout promoted anoikis resistance and in vivo tumorigenicity in TNBC cells.

(A) DNA sequencing validation of PTPN14 knockout. Three independent experiments were performed. (B) Validation of PTPN14 knockout in BT549 cells through western blotting. Three independent experiments were performed. (C) Cell proliferation of the control and PTPN14-knockout BT549 cells in monolayer adherent culture was assessed every 24 hours for 5 days using the CCK-8 assay (n=3). Student’s t-test, ***p < 0.001. (D) The counts of living cells for both control BT549 cells and PTPN14-knockout BT549 cells were determined after culturing under ULA condition for 7 days (n=3). Student’s t-test, **p < 0.01. (E) Representative images of colony formation assays in control MDA-MB-231 cells and PTPN14-knockout MDA-MB-231 cells (left), along with statistical summaries of the results from three independent experiments (right). Student’s t-test. (F) Western blot analysis was performed to assess the phosphorylation of AKT and ERK in PTPN14-KO BT549 cells after 12 hours of culture under ULA condition (left), along with statistical summaries of the results from three independent experiments (right). Student’s t-test, *p < 0.05, **p < 0.01. (G) and (H) Western blot analysis was employed to assess the protein expression levels in each group of cells. Three independent experiments were performed. (I) Representative images of 3D invasion analysis of control and PTPN14-KO MDA-MB-231 cells, with white arrows indicating protrusions. Three independent experiments were performed. Scale bar: 100 μm. (J) The body weight of the two groups of mice was recorded from tumors implantation to the experiment's end. (K) Representative H&E staining images, CD31 IHC images, Ki-67 IHC images and PTPN14 IHC images of two groups of tumor sections (n=3). Scale bar: 100 μm.

Figure S3

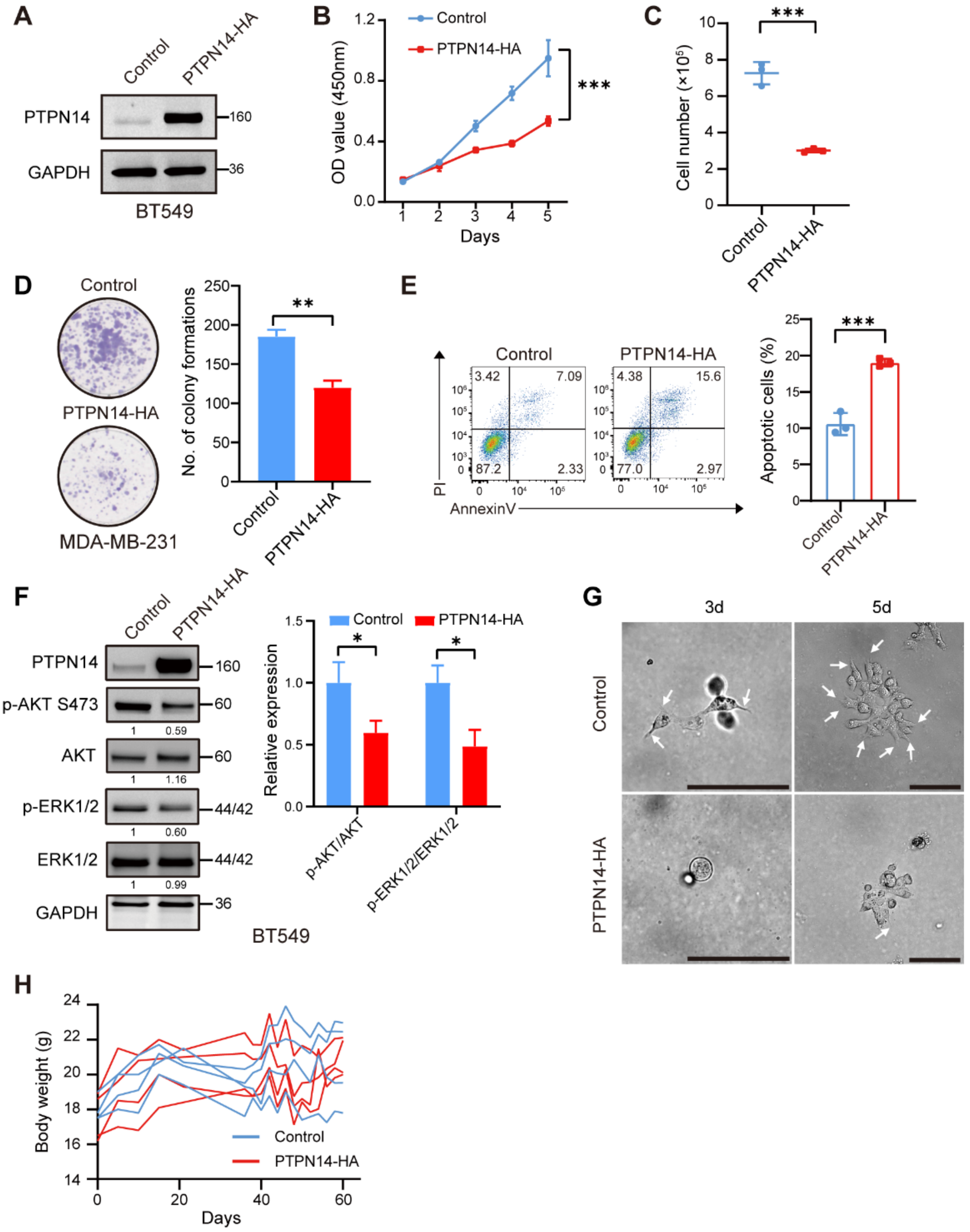
A
Control
PTPN14-HA
PTPN14
GAPDH
160
36
BT549
B
OD value (450nm)
Days
Control
PTPN14-HA
***
C
Cell number (×10⁵)
***
Control
PTPN14-HA
D
Control
PTPN14-HA
MDA-MB-231
No. of colony formations
**
Control
PTPN14-HA
E
Control
PTPN14-HA
3.42
7.09
87.2
2.33
4.38
15.6
77.0
2.97
PI
AnnexinV
Apoptotic cells (%)
***
Control
PTPN14-HA
F
Control
PTPN14-HA
PTPN14
p-AKT S473
AKT
p-ERK1/2
ERK1/2
GAPDH
160
60
60
44/42
44/42
36
1
0.59
1
1.16
1
0.60
1
0.99
Control
PTPN14-HA
Relative expression
*
*
p-AKT/AKT
p-ERK1/2/ERK1/2
BT549
G
3d
5d
Control
PTPN14-HA
H
Body weight (g)
Days
Control
PTPN14-HA

Figure S3-continued

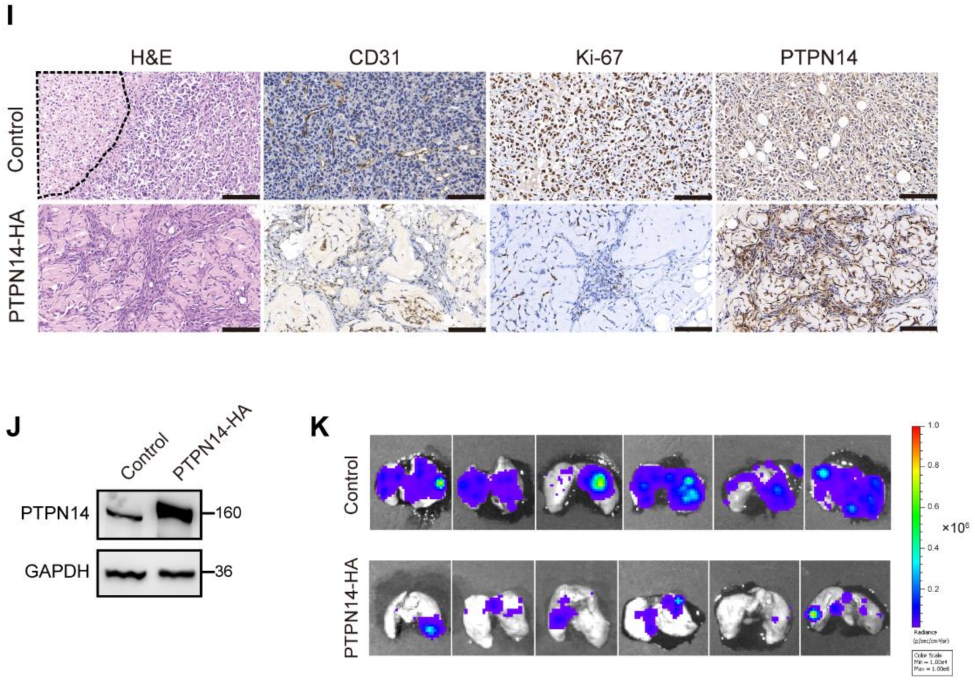

Figure S3. PTPN14 overexpression induced anoikis and suppressed in vivo tumorigenicity and pulmonary metastasis in TNBC cells.

(A) Validation of PTPN14 overexpression in BT549 cells through western blotting. Three independent experiments were performed. (B) Cell proliferation of the control and PTPN14-OE BT549 cells in monolayer adherent culture was assessed every 24 hours for 5 days using the CCK-8 assay (n=3). Student's t-test, ***p < 0.001. (C) The counts of living cells for both control BT549 cells and PTPN14-OE BT549 cells were determined after culturing under ULA condition for 7 days (n=3). Student's t-test, ***p < 0.001. (D) Representative images of colony formation assays in control MDA-MB-231 cells and PTPN14-OE MDA-MB-231 cells (left), along with statistical summaries of the results from three independent experiments (right). Student's t-test, **p < 0.01. (E) After culturing for 3 days under ULA condition, cell apoptosis was measured by flow cytometry in both control MDA-MB-231 cells and PTPN14-OE MDA-MB-231 cells. Left, representative flow cytometry result plots; right, statistical summaries of the results from three independent experiments. Student's t-test, ***p < 0.001. (F) Western blot analysis was performed to assess the phosphorylation of AKT and ERK in PTPN14-OE BT549 cells after 12 hours of culture under ULA condition (left), along with statistical summaries of the results from three independent experiments (right). Student's t-test, *p < 0.05. (G) Representative images of 3D

invasion analysis of control and PTPN14-OE MDA-MB-231 cells, with white arrows indicating protrusions. Three independent experiments were performed. Scale bar: 100 µm. (H) The body weight of the two groups of mice was recorded from tumor implantation to the experiment's end. (I) Representative H&E staining images, CD31 IHC images, Ki-67 IHC images and PTPN14 IHC images of two groups of tumor sections (n=3). Scale bar: 100 µm. (J) Validation of the expression levels of PTPN14 in the two groups of MDA-MB-231-Luc cells used for the tail vein metastasis model by western blotting. Three independent experiments were performed. (K) Ex vivo lung imaging of mice from two groups on the 41st day post-injection (n=6).

Figure S4

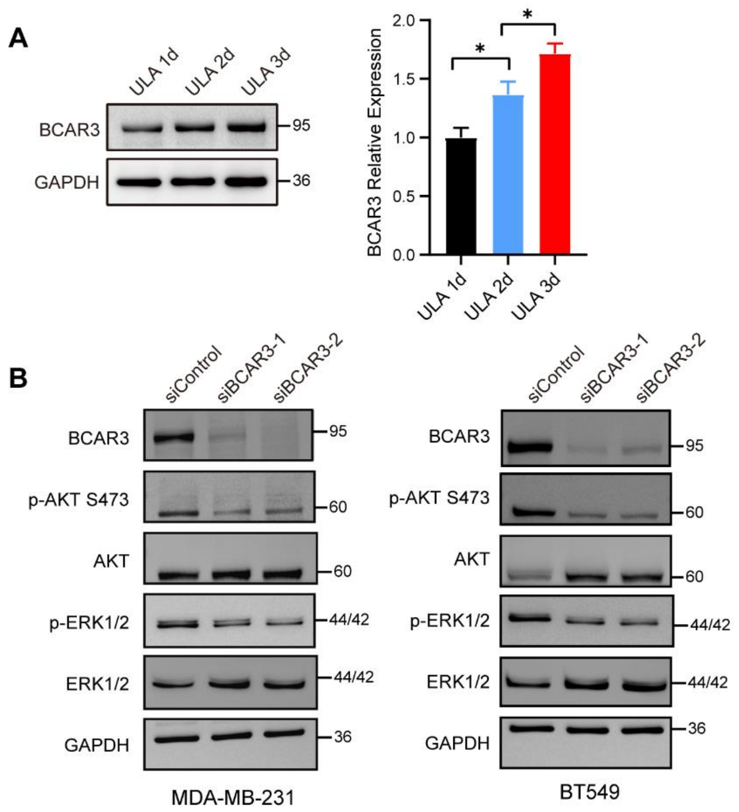

Figure S4. BCAR3 identified as a substrate of PTPN14.

(A) Western blot analysis of BCAR3 expression in MDA-MB-231 cells cultured under ULA condition for 1 day, 2 days, and 3 days (n=3). Tukey's multiple comparisons test follow one-way ANOVA, *p < 0.05. (B) Western blot analysis was performed to assess the phosphorylation of

AKT and ERK following BCAR3 knockdown in MDA-MB-231 and BT549 cells. Three independent experiments were performed.

Figure S5

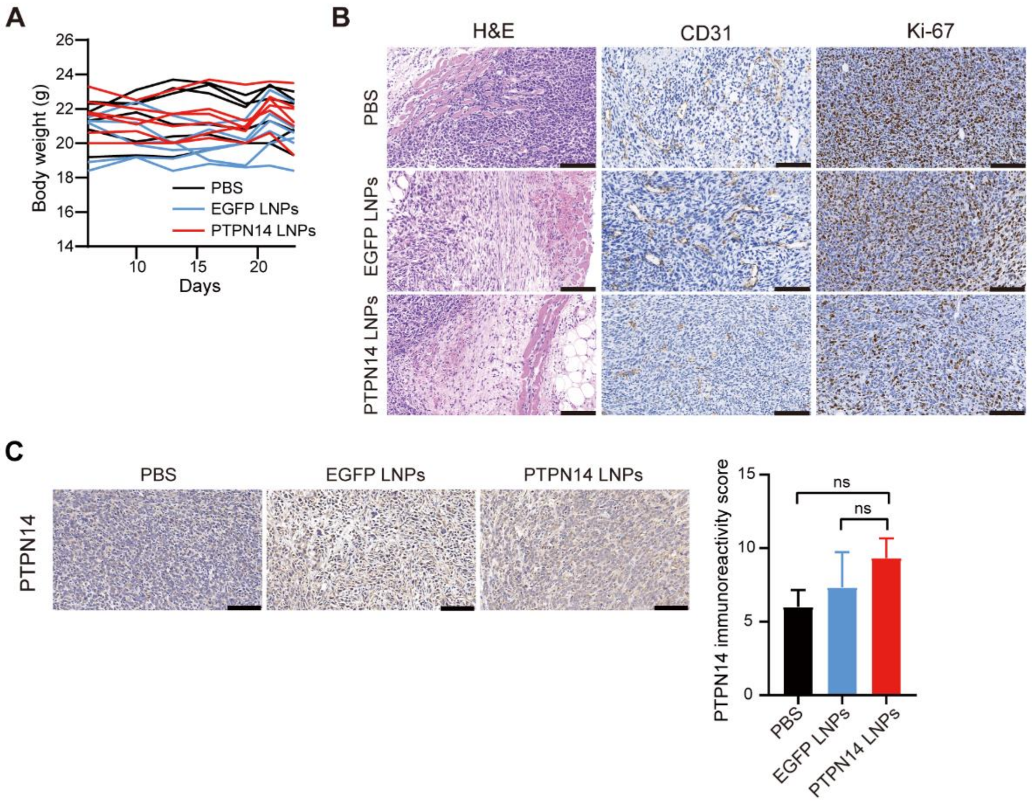

Figure S5-continued

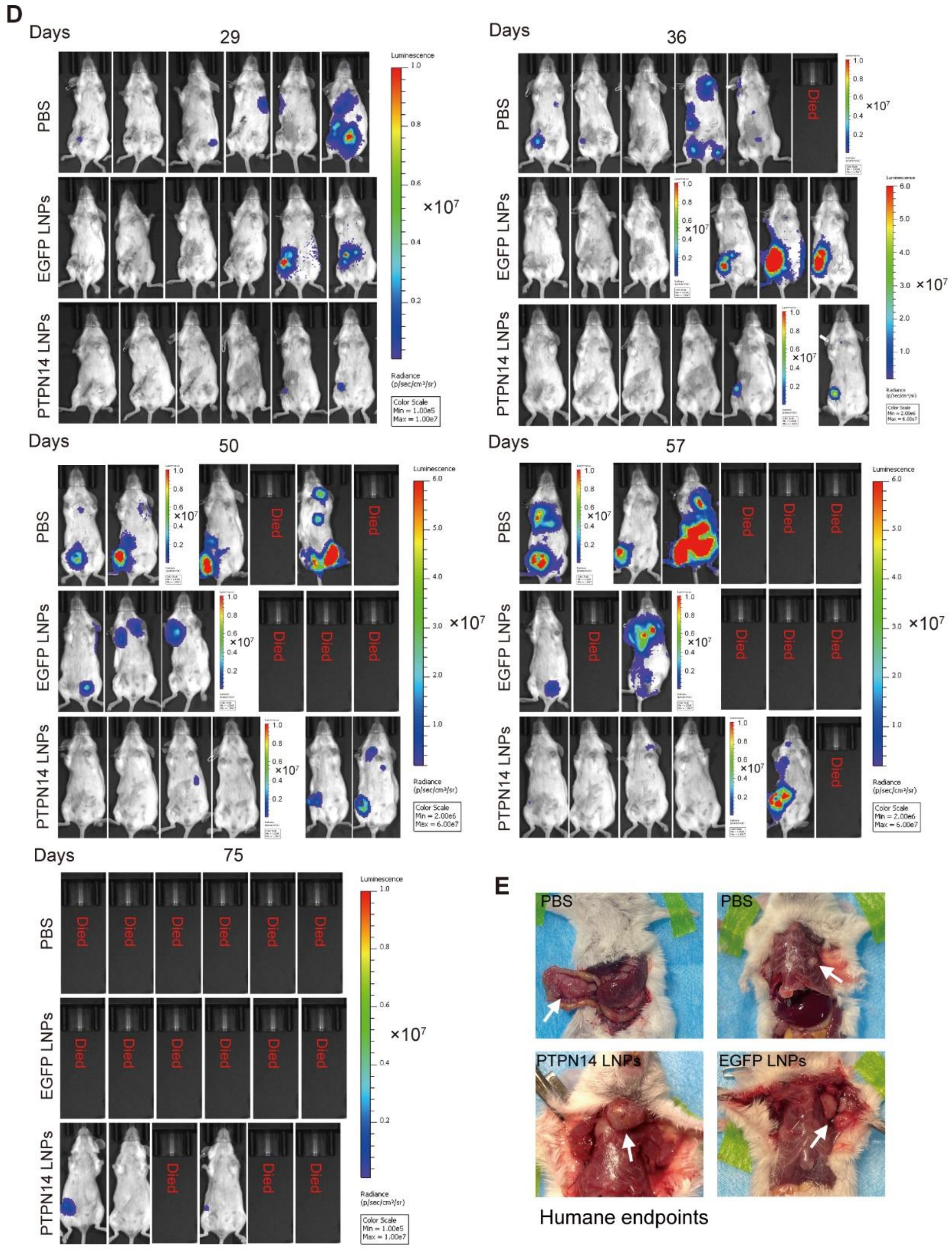

Figure S5. Effects of PTPN14 mRNA LNPs on the growth and metastasis of 4T1 tumors. (A) Individual mouse body weight curves during the mRNA LNPs treatment period. (B) Representative images of H&E staining, CD31 IHC and Ki-67 IHC in orthotopic tumor tissue sections from each group of mice (n=3). Scale bar: 100 μm. (C) Representative PTPN14 IHC images (left), along with quantification of the PTPN14 IHC staining results (right) (n=3). Scale

bar: 100 μm. Tukey's multiple comparisons test following one-way ANOVA. (D) In vivo imaging of mice from each group on the 29th, 36th, 50th, 57th, and 75th days post-4T1 tumor inoculation. (E) Representative images of atypical metastatic sites in the 4T1 tumor model, with the top left showing mesenteric metastasis, top right showing chest wall metastasis, bottom left showing thyroid metastasis, and bottom right showing lymph node metastasis with lymph node fusion.
